# Metabolic Profiling of Interspecies Interactions During Sessile Bacterial Cultivation Reveals Growth and Sporulation Induction in *Paenibacillus amylolyticus* in Response to *Xanthomonas retroflexus*

**DOI:** 10.3389/fcimb.2022.805473

**Published:** 2022-03-29

**Authors:** Jakob Herschend, Madeleine Ernst, Klaus Koren, Alexey V. Melnik, Ricardo R. da Silva, Henriette L. Røder, Zacharias B. V. Damholt, Per Hägglund, Birte Svensson, Søren J. Sørensen, Michael Kühl, Pieter C. Dorrestein, Mette Burmølle

**Affiliations:** ^1^ Section of Microbiology, Department of Biology, University of Copenhagen, Copenhagen, Denmark; ^2^ Collaborative Mass Spectrometry Innovation Center, Skaggs School of Pharmacy and Pharmaceutical Sciences, University of California, San Diego, La Jolla, CA, United States; ^3^ Section for Clinical Mass Spectrometry, Danish Center for Neonatal Screening, Department of Congenital Disorders, Statens Serum Institut, Copenhagen, Denmark; ^4^ Aarhus University Centre for Water Technology (WATEC), Department of Biology, Aarhus University, Aarhus, Denmark; ^5^ Department of Biotechnology and Biomedicine, Technical University of Denmark, Kgs Lyngby, Denmark; ^6^ Marine Biological Section, Department of Biology, University of Copenhagen, Helsingør, Denmark

**Keywords:** pH stabilization, omics, sporulation, microsensor, interspecies interactions

## Abstract

The toolbox available for microbiologists to study interspecies interactions is rapidly growing, and with continuously more advanced instruments, we are able to expand our knowledge on establishment and function of microbial communities. However, unravelling molecular interspecies interactions in complex biological systems remains a challenge, and interactions are therefore often studied in simplified communities. Here we perform an in-depth characterization of an observed interspecies interaction between two co-isolated bacteria, *Xanthomonas retroflexus* and *Paenibacillus amylolyticus*. Using microsensor measurements for mapping the chemical environment, we show how *X. retroflexus* promoted an alkalization of its local environment through degradation of amino acids and release of ammonia. When the two species were grown in proximity, the modified local environment induced a morphological change and growth of *P. amylolyticus* followed by sporulation. 2D spatial metabolomics enabled visualization and mapping of the degradation of oligopeptide structures by *X. retroflexus* and morphological changes of *P. amylolyticus* through e.g. the release of membrane-associated metabolites. Proteome analysis and microscopy were used to validate the shift from vegetative growth towards sporulation. In summary, we demonstrate how environmental profiling by combined application of microsensor, microscopy, metabolomics and proteomics approaches can reveal growth and sporulation promoting effects resulting from interspecies interactions.

## Introduction

Natural microbial communities often host a plethora of different species, where environmental factors such as pH, O_2_, temperature and water availability are known to dictate community diversity and largely drive its development through sharp environmental gradients (de Wit and Bouvier, 2006; Rousk et al., 2009; He et al., 2012; Becking and Canfield, 2015; Cao et al., 2016). However, several studies have also shown that interspecies interactions can significantly affect community growth, composition, activity, and function (Burmølle et al., 2014; Madsen et al., 2018). Understanding inter-species interactions is therefore important for managing microbial communities in environmental and industrial settings. Direct analysis and identification of inter-species interactions in natural communities is often hampered by community complexity leading to methodological and technical restraints (VerBerkmoes et al., 2009). On the other hand, studying individual community members outside of their community context rarely enables prediction of interspecies interactions, as these are often induced by other species and lead to community intrinsic properties caused by the community context, providing unexpected functions or growth patterns for mixed-species communities (Madsen et al., 2018). Co-culture studies using simpler, pre-defined model communities are therefore often applied to unravel specific microbial interactions. This has for example allowed the identification of numerous types of both intra- and inter-species interactions, such as i) cooperative cross-feeding on secreted by- or waste-products (Rhee et al., 2000; Pande et al., 2014; Pande et al., 2016), ii) cross-protection from antibiotics facilitated by resistant strains protecting non-resistant strains (Lee et al., 2014; Yurtsev et al., 2016) or iii) through competition by toxin secretion (Kerr et al., 2002).

Previous work on a well-described model community constructed from four co-isolated bacterial species from Danish soil (de la Cruz-Perera et al., 2013), containing *Stenotrophomonas rhizophila*, *Xanthomonas retroflexus*, *Microbacterium oxydans* and *Paenibacillus amylolyticus*, has shown how different interspecies interactions can drive synergistic community growth. For example, the synergistic biofilm formation from this community (Ren et al., 2015) has previously been linked to cross-feeding on amino acids (Hansen et al., 2017; Herschend et al., 2017), unique spatial organization (Liu et al., 2017; Liu et al., 2018) and environmental pH stabilization (Herschend et al., 2018). In line with this, Ratzke and Gore (2018) have shown that the outcome of pH-driven interactions can be modelled when the pH drift and pH growth optimum is known for the interacting partners. Here pH stabilization was defined as a scenario where two bacteria, which on their own would create a detrimental pH environment, can co-exist by canceling each other’s pH drift. A similar type of pH interaction was also shown to occur between two members of our previously studied, four species model community, *X. retroflexus* and *P. amylolyticus* (Herschend et al., 2018), and was identified as a strong contributor to the synergy observed in this community. Specifically, we showed that the cultivation of a *P. amylolyticus* colony in proximity to a colony of *X. retroflexus* caused a morphological change in the *P. amylolyticus* colony and that this change was likely driven by pH stabilization of the environment through the metabolic activity of *X. retroflexus.* In the present study, we investigate the metabolic and chemical underpinnings of the pH-related interaction of *X. retroflexus* and *P. amylolyticus* in greater depth using a combination of microsensor measurements, metabolomics and proteomics approaches. Microsensor and ammonia measurements tracked the steep chemical gradients emerging during the interaction, revealing the interplay between ammonia release and pH drift leading to altered growth potential of *P. amylolyticus*. Subsequently, we used mass spectrometry and molecular networking analysis through the Global Natural Products Social Molecular Networking (GNPS) platform (Watrous et al., 2012; Wang et al., 2016) to characterize the bacterial interaction at a molecular level and, in combination with proteomics, show how this interaction led to induced growth followed by sporulation in *P. amylolyticus.*

## Results and Discussion

### Morphological Characterization of the Two Species Interaction

**Figure 1:**
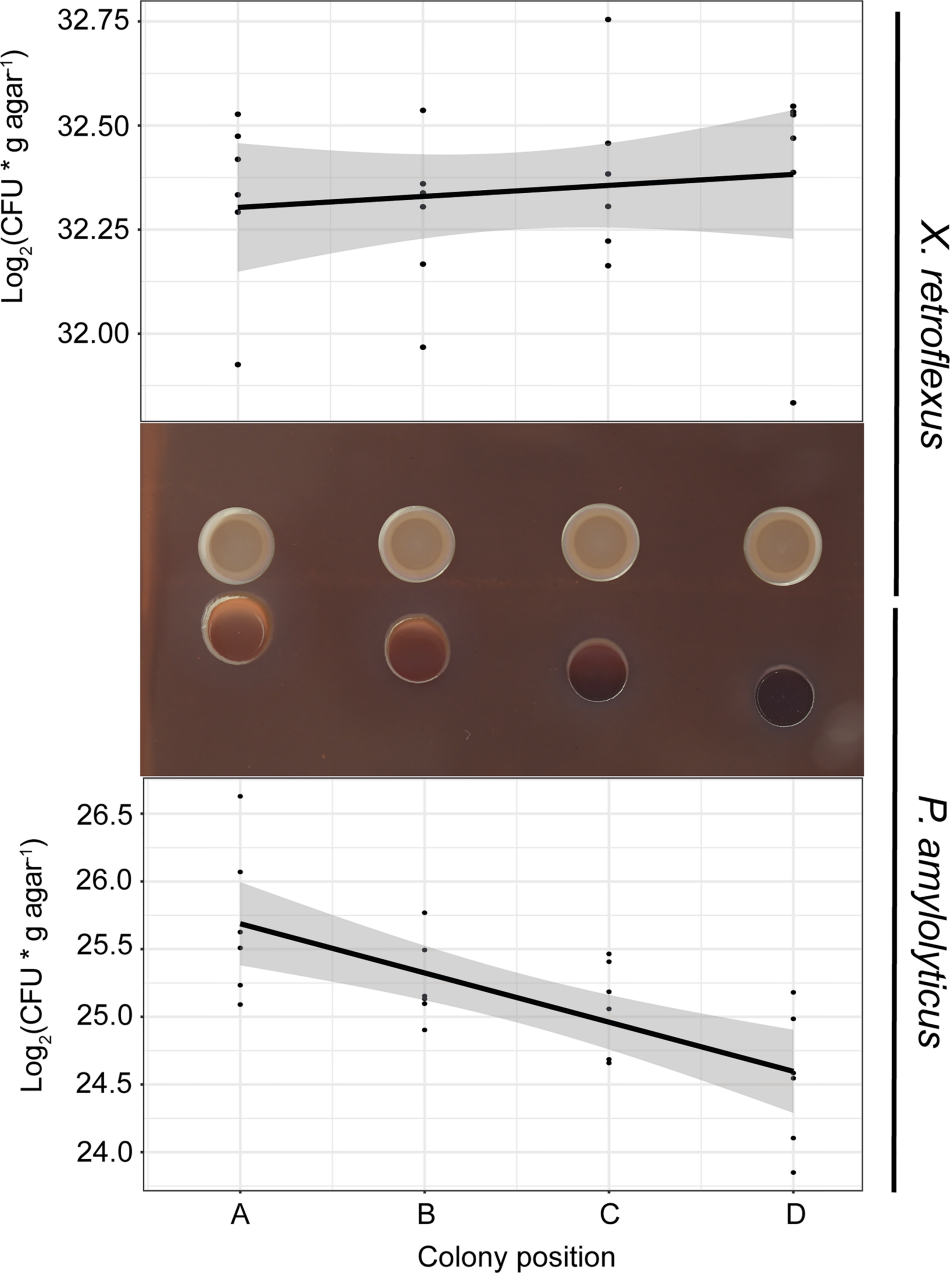
Bacterial growth as a function of distance (position **A–D**) between colonies of *X. retroflexus* and *P. amylolyticus*. After 48 hrs of growth, *X. retroflexus* induced colony growth and a change in colony morphology of *P. amylolyticus* with reduced distance between the colonies when cultured on 50% Congo red agar plates. The colony of *P. amylolyticus* became increasingly more red with closer proximity, and the periphery of the *P. amylolyticus* colony became disordered in texture and displayed directional growth towards *X. retroflexus*. Log2(CFU* g agar-1) of *P. amylolyticus* was significantly lower with increasing distance, as calculated by a linear model (Coefficient; -0.36, R2 = 0.47, p-value = 0.00014). No significant linear trend was observed for *X. retroflexus* colonies (Coefficient; 0.026, R2 = -0.025, p-value = 0.5131).

*P. amylolyticus* and *X. retroflexus* were spotted on Congo red agar plates with increasing distance between colonies (**Figure 1**), to morphologically characterize the interaction. Interactions examined include direct (metabolite/compound produced by one strain directly affecting the other) as well as indirect interactions (metabolite/compound produced by one strain altering medium composition with subsequent effects on the other). In this experimental design, direct and indirect interactions are not distinguishable.

After 48 hrs of incubation, the colony of *P. amylolyticus* was visually affected by the presence of *X. retroflexus*. With closer proximity to *X. retroflexus*, *P. amylolyticus* displayed i) color-change from dark brown to red, ii) disordered texture of the colony facing *X. retroflexus*, and iii) visually noticeable directional growth towards *X. retroflexus*. The color change of the colony is likely a result of altered binding of Congo red and coomassie brilliant blue G250, where Congo red binds various carbohydrate matrix components and coomassie binds to various protein components in the matrix. Directional growth of *P. amylolyticus* indicated attraction towards *X. retroflexus*, likely caused by *X. retroflexus* influencing the local environment, making it more favorable by e.g. increasing pH as previously observed (Herschend et al., 2018), favorably modifying the growth medium, and/or by secretion of metabolites with a nutritional value. Counts of colony forming units (CFU) of *P. amylolyticus* colonies revealed that proximity to *X. retroflexus* (**Figure 1**) increased CFU counts, with a significantly negative linear relationship between distance and cell counts (Coefficient; -0.36, R^2^ = 0.47, p-value = 0.00014; linear model). No change in morphology was observed for *X. retroflexus* and no correlation was found between distance and cell counts (Coefficient; 0.026, R^2^ = -0.025, p-value = 0.5131), indicating that *X. retroflexus* was not affected by the proximity of *P. amylolyticus.*

### Chemical Characterization of the Two-Species Interaction

**Figure 2:**
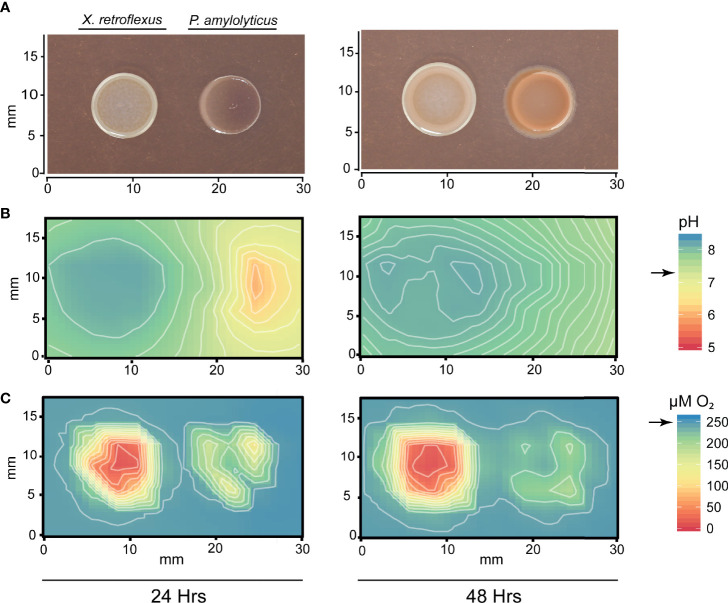
Mapping of pH and O_2_ concentration around colonies of *X. retroflexus* and *P. amylolyticus*. **(A)** Colony morphology showing the changed morphology of *P. amylolyticus* at 24 and 48 hrs. **(B)** Distribution of pH around the bacterial colonies showing a change of pH in the interaction zone. *X. retroflexus* was alkalizing the interaction zone from 24 hrs to 48 hrs. At 24 hrs, the backside of the *P. amylolyticus* colony was acidic, with a pH as low as 5-6. At 48 hrs the pH around the *P. amylolyticus* was shifted to approx. pH 7 from the pH drift caused by *X. retroflexus*. Black arrow on scale bar indicates pH of TSA. **(C)** Distribution of O_2_ concentration around the bacterial colonies showing a high O_2_ consumption of *X. retroflexus* and lower consumption from *P. amylolyticus*. Oxygen consumption of *P. amylolyticus* seemed restricted to the periphery of the colony at both 24 and 48 hrs, however with a lower consumption at 48 hrs. Black arrow on scale bar indicates oxygen level in TSA without inoculation of bacteria. Combined alkalization and high oxygen consumption of *X. retroflexus* indicate a respiratory metabolism that is likely based on degradation of amino acids. In contrast, acidification and low oxygen consumption indicate a fermentative metabolism for *P. amylolyticus*. Plots of single species interactions are available for *X. retroflexus* in **Figure S1** and for *P. amylolyticus* in **Figure S2**.

To investigate the local chemical environment of the two-species interaction, we mapped the O_2_ concentration and pH over and in between the bacterial colonies using microsensors. **Figure 2** shows changes in colony morphology, pH and O_2_ environment across the interaction zone, after one and two days of growth respectively. The area around and beneath the *X. retroflexus* colony became increasingly alkaline from 24 to 48h, changing the entire interaction zone pH to a level above that of the pH of TSA. However, at 24 hrs, *P. amylolyticus* had contrarily acidified the environment. *X. retroflexus* was actively growing and consuming O_2_, both at 24 and 48 hrs, while O_2_ consumption of *P. amylolyticus* did not deprive the agar of O_2_ to the same extent. Oxygen consumption of *P. amylolyticus* was observed to be higher at 24 hrs and mostly located in the periphery of the colony, potentially reducing growth at 48 hrs. Control plates with single species interactions validated environmental alkalization and high levels of O_2_ consumption for *X. retroflexus* (**Figure S1**). In contrast, environmental acidification and reduced O_2_ consumption over time was observed for *P. amylolyticus* (**Figure S2**). This pattern confirmed previous observations regarding pH driven interactions of these two species in a four-species model consortium (Herschend et al., 2018).

**Figure 3:**
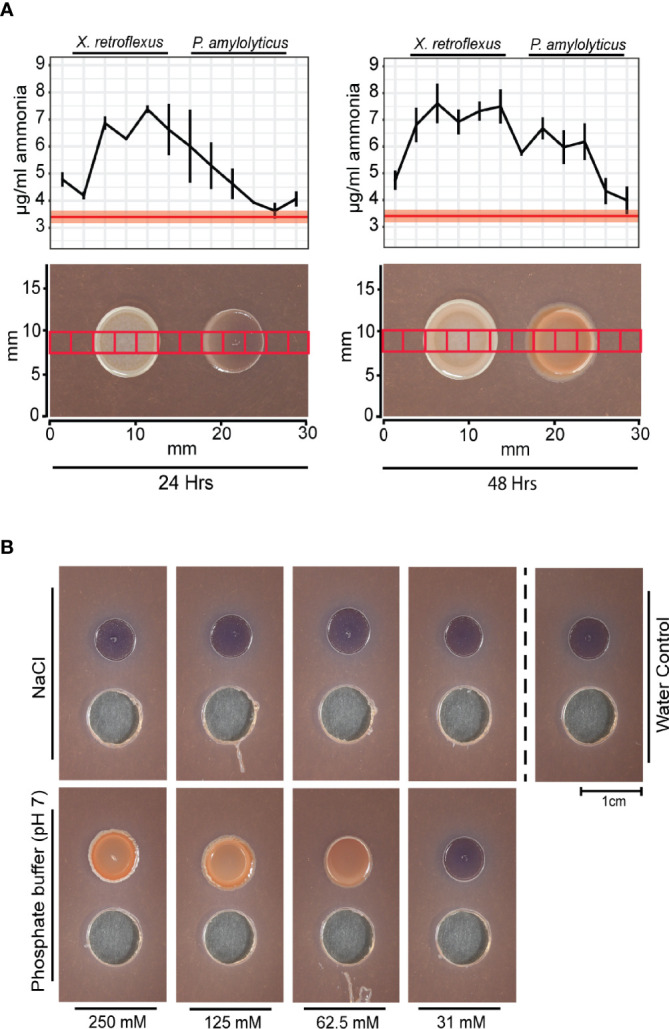
The pH stabilized interaction of *X. retroflexus* and *P. amylolyticus*. **(A)** Ammonia profiles measured over the interaction zone of *X. retroflexus* and *P. amylolyticus*. 2.5 by 2.5 mm agar plugs were excised from the interaction zone according to the red squares overlayed in the morphology panel. The ammonia release was measured from each excised agar plug. Red line in the plot shows the mean ammonia concentration in control agar (50% TSA), with the red transparent ribbon indicating standard error of the mean (n=3). Black line represents mean ammonia concentration across the interaction zone, with horizontal bars indicating standard error of measured ammonia (n=3). At 24 hrs, ammonia released from *X. retroflexus* formed a gradient across the *P. amylolyticus* colony. At 48 hrs the amount of ammonia increased in the interaction zone as well as over the *P. amylolyticus* colony. The images show the morphological changes of P. amylolyticus occurring from 24 hrs to 48 hrs. **(B)** Effect of pH buffering on the growth of *P. amylolyticus*. *P. amylolyticus* was cultivated for 48 hrs adjacent to wells containing 100 µL NaCl or phosphate buffer (pH 7) in concentrations ranging from 250-31 mM and with sterile MilliQ water as control. A morphological change of the *P. amylolyticus* colony was observed when adding buffer with increasing buffer capacity, whereas a similar salt concentration did not induce the morphological change. Individual plots with ammonia profiles for *X. retroflexus* and *P. amylolyticus* are available as **Figure S4**.

Metabolic catabolism of amino acids through oxidative deamination leads to production of ammonia, which can alkalize the environment by sequestering protons. The high oxygen consumption from *X. retroflexus* could support such metabolic activity. In contrast, fermentation of glucose can lead to acidification of the environment. However, there are other metabolic pathways that can also lead to environmental alkalization, e.g. the arginine and agmatine deiminase systems, previously described as important in oral biofilms. Commensal bacteria in human oral biofilms need to repeatedly alkalize their environment to exclude competitors that thrive at low pH and to cope with the repeating acidification occurring as a response to degradation of carbohydrate structures from the human diet (Burne and Marquis, 2000; Clancy et al., 2000). Both arginine (Burne and Marquis, 2000) and agmatine deiminase (Griswold et al., 2006) pathways are catabolic processes known from e.g. *Streptococcus* spp. as a mean to cope with acidification in oral biofilms and generate ATP. By metabolizing arginine or agmatine, *Streptococcus* spp. produces ammonia as by-product that can help maintain pH homeostasis. Metabolic profiling in Hugh and Leifson’s media supported alkalization through catabolism of amino acids by *X. retroflexus* and anaerobic glucose fermentation by *P. amylolyticus* (**Figure S3**). To validate ammonia release, ammonia was measured in agar plugs excised across the interaction zone, according to the grid layout used for mapping the chemical environment (**Figure 3A**). The ammonia profile transecting the dual-species interaction zone indicated an increased amount of ammonia as compared to blank agar. At 24 hrs, a gradient of ammonia was found around the *X. retroflexus* colony, which then traversed the *P. amylolyticus* colony at 48 hrs. Growth of *X. retroflexus* alone showed ammonia build-up around the colony, whereas no ammonia production was observed for *P. amylolyticus* when cultured alone (**Figure S4**).

To build upon the hypothesis of pH stabilization of the external environment as a facilitator for the morphological change, *P. amylolyticus* was cultivated with addition of pH adjusted phosphate buffer at pH 7. Addition of a >31 mM phosphate buffer caused a visual morphological change and increased density of the colony resembling that observed during interaction with *X. retroflexus*. A similar response was not induced by adding comparable concentrations of salt, thus indicating that the response was caused by pH stabilization (**Figure 3B**). Optimal pH growth range for *P. amylolyticus* has previously been observed as pH 6-8 (Herschend et al., 2018).

### Metabolomics Characterization of Two-Species Interaction

To further characterize chemical changes within the interaction zone of *P. amylolyticus* and *X. retroflexus* at a molecular level, agar plugs across the interaction zone were collected and subjected to liquid chromatography – tandem mass spectrometry (LC-MS/MS) based metabolomics. Plugs were collected according to the grid structure used for mapping the chemical environment, see **Figure S6** for metabolomics workflow. A mixed solvent extraction protocol was applied to enhance metabolite extraction and diversity. Metabolites were extracted from single- and dual-species interaction zones at 24 and 48 hrs of incubation. Chemical structural information of detected molecules was retrieved by mass spectral molecular networks through Global Natural Products Social Molecular Networking (GNPS) (Watrous et al., 2012; Wang et al., 2016). In short, each node in the network corresponds to a unique structural feature (unique metabolite), association between nodes is based on structural similarity and each node is color-coded based on which sample group the structural feature was observed in, e.g. agar control or *P. amylolyticus* single species samples. An extensive overview of the workflow is available in Material and Methods.

Most of the nodes were found in the agar control samples (3629 nodes), whereas only 1506 nodes were uniquely found in *X. retroflexus*, *P. amylolyticus* or the interaction zone. Of the 1506 nodes, 84 nodes were uniquely detected in the dual-species interaction zone, 1075 nodes were shared between the dual-species and single-species interaction zones and 347 nodes were uniquely allocated in the single species controls (single-species, **Table S1**).

In short, feature annotation of the nodes was performed using a combination of Network Annotation Propagation (da Silva et al., 2018), Dereplicator (Mohimani et al., 2017), ClassyFire (Feunang et al., 2016) and MolNetEnhancer (Ernst et al., 2019), see *Material and Methods* for a detailed description of the workflow. Additionally, **Table S2** summarises counts of molecular families, and **Table S3** summarises putative annotation yield through the MolNetEnhancer workflow and GNPS.

**Figure 4:**
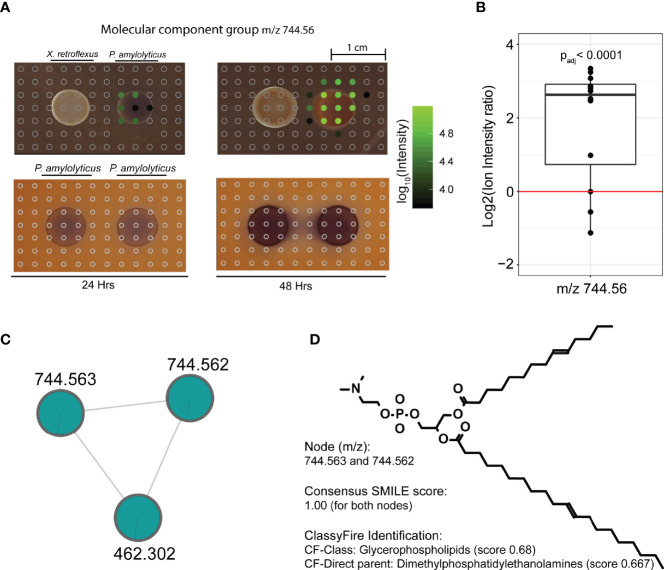
Spatial distribution of a metabolite feature, unique to the inter-species interaction, across the bacterial colonies and their interaction zone. **(A)** Spatial distribution of molecular feature with m/z 744.56, unique to the interspecies interaction. Circles indicate positioning of excised agar plugs across the interaction zone. Empty grey circles refer to spots where the metabolite was not detected. Filled circles refer to plugs where the metabolite was detected, and color from black to green refers to Log10(MS1 ion intensity). Three data files were lost during analysis, which corresponds to the blank area without circles in the interaction zone. At 24 hrs the metabolite was only detected in the periphery of the *P. amylolyticus* colony facing the *X. retroflexus* colony, whereas it was highly abundant across the entire *P. amylolyticus* colony at 48 hrs. **(B)** Ion intensity plot of the plugs covering the 48 hrs colony of the interacting *P. amylolyticus*, as compared to the non-interacting 48 hrs colony. For the interacting colony, MS1 ion intensities were divided by 3 to account for the higher observed cell count. Even when accounting for increased number of cells in the interacting colony and an inferred artificial detection limit, metabolite m/z 744.56 was significantly more abundant in the interacting colony. **(C)** Molecular family containing mass spectral feature with m/z 744.56, for statistical analysis unique mass spectral features likely split during preprocessing with m/z 744.563 and m/z 744.562 were merged. The two nodes grouped together with an additional molecular feature, represented in **Figure S8**. **(D)** *In silico* putative structure annotation identified the molecule as a glycerophospholipid.

To understand the morphological change of *P. amylolyticus* at a molecular level, we assessed the spatial distribution of the *in silico* annotated structures unique to the single or dual-species interaction zone, comprising 12 molecular families. Prior to visualisation, nodes from the same molecular family with similar MS2 spectra were collapsed into a single feature to enable better visualisation, see *Materials and Methods* and **Figure S7**. A distribution summary of molecular families is available from **Table S4**. Of the 12 families, 3 molecular families (referred to by their component index [CI]; CI 113, CI 114 and CI 159) showed a unique spatial distribution across the *P. amylolyticus* colony, with two molecular families being unique to the dual-species interaction zone. **Figure 4** exemplifies spatial distribution of a molecule from molecular family with CI 114. At 24 hrs the mass spectral molecular feature with a *m/z* of 744.56 was detected only in the periphery of the *P. amylolyticus* colony facing the *X. retroflexus*, whereas it was highly abundant across the entire *P. amylolyticus* colony at 48 hrs. *In silico* putative structure annotation annotated this mass spectral molecular feature as a glycerophospholipid. Glycerophospholipids are common components of bacterial membranes and their release to the environment can be explained by cell wall disruption e.g. from cell death, sporulation or production of membrane vesicles known to facilitate the release of proteins, including toxins and other virulence factors (Anand and Chaudhuri, 2016). However, changes in the pH environment can also lead to chemical changes in glycerophospholipid in the bacterial membranes e.g. changing the ratios of unsaturated to saturated, *cis* to *trans* unsaturated, and branched to unbranched fatty acids (Guan and Liu, 2020). Hence, the emergence of the annotated glycerophospholipid could be a response to the interaction itself and/or the changed pH environment, resulting in cell wall disruption or reorganization, or vesicle production. A similar pattern was also observed for the other feature in CI 114 with *m/z* 462.302 (**Figure S8**). For, e.g. CI 114, the molecular feature was uniquely observed in the interacting colony and could as such be argued to be a direct response to the interaction with *X. retroflexus*. However, as the interaction with *X. retroflexus* also resulted in enhanced cell numbers of *P. amylolyticus* the observed feature could also be a response to increased cell counts - and not to a phenotypic change. To support that the molecular feature was a response to altered phenotypic behaviour, the level of MS1 ion intensity of several sampling points across the interacting *P. amylolyticus* colony was compared to that of the non-interacting colony, see *Materials and Methods*. Even when inferring an artificial detection limit and accounting for exponential compound production at the higher CFU level in the interaction colony, the level of compound intensities across the interacting colony was still significantly higher (p_adj_ = 0.00645). The same trend was observed for the feature with *m/z* 462.302 within CI 114 (**Figure S8**). This observation supported that glycerophospholipid structural analogues were released from the interacting colony of *P. amylolyticus* as a response to its interaction with *X. retroflexus*. In further support, molecular features with *m/z* 752.539, 766.543 and 778.546 from CI 113 were putatively annotated as glycerophospholipids, and although they were observed in the *P. amylolyticus* single-species samples (**Figure S9**) their level was higher in the interacting colony – the difference was significant for *m/z* 766.543 and *m/z* 778.546 (p_adj_ = 0.0002 and p_adj_ = 0.003, respectively). For the two molecular features with *m/z* 593.3 and 607.379 in CI 159, MolNetEnhancer suggested prenol lipid structural analogues, although with a very low score (0.348) (**Figure S10**). Some types of prenol lipids are known to be associated to bacterial membranes, such as e.g. bactoprenols and hopanoids. The two features were also significantly more abundant in the interacting colony (p_adj_ < 0.0001 for both *m/z* 593.3 and 607.379). These two features were even more abundant than features from CI 113 and 114. Among prenol lipids, hopanoids are thought to be prokaryotic equivalent of eukaryotic sterols, and hence several studies have shown that hopanoids are important to maintain membrane integrity in stressful conditions, e.g. under pH related stress (Welander et al., 2009; Schmerk et al., 2011; Sáenz et al., 2015).

An additional number of 20 molecular features from the self-looping group also showed unique distribution around the colony of *P. amylolyticus* and many of these were significantly more abundant than the artificial detection limit after accounting for increased cell counts in the interacting colony, see **Figures S11–S13**.

A major drawback of *in silico* putative structure annotation is the uncertainty concerning the correct structure (da Silva et al., 2018) and results discussed here should thus be interpreted with caution. Absolute chemical structure identification would require compound isolation and application of spectroscopic methods, which is out of scope of the current study.

*X. retroflexus* did not yield any molecular features hinting towards a radically changed and unique phenotype when growing near *P. amylolyticus.* Molecular families unique to *X. retroflexus* were observed, but for both the interacting and non-interacting colony, e.g. CI 70, 168, 277, 347, 352 and 369. These molecular features were mostly related to putative membrane associated components, e.g. prenol lipids (CI 347) and glycerophospholipids (CI 362). Interestingly some of these molecular features were significantly increased for the interacting colony (p_adj_ = 0.0002 for CI 347; p_adj_ < 0.0001 for CI 352; p_adj_ = 0.006 for CI 362), suggesting that *X. retroflexus* might adjust its membrane composition dependent on the presence of *P. amylolyticus*. In support, of alkalization by catabolism of amino acids by *X. retroflexus*, a number of oligo-peptides showed a spatial reduction around the colony of over time (**Figure S14**).

#### Phenotypic Changes in *P. amylolyticus*

**Figure 5:**
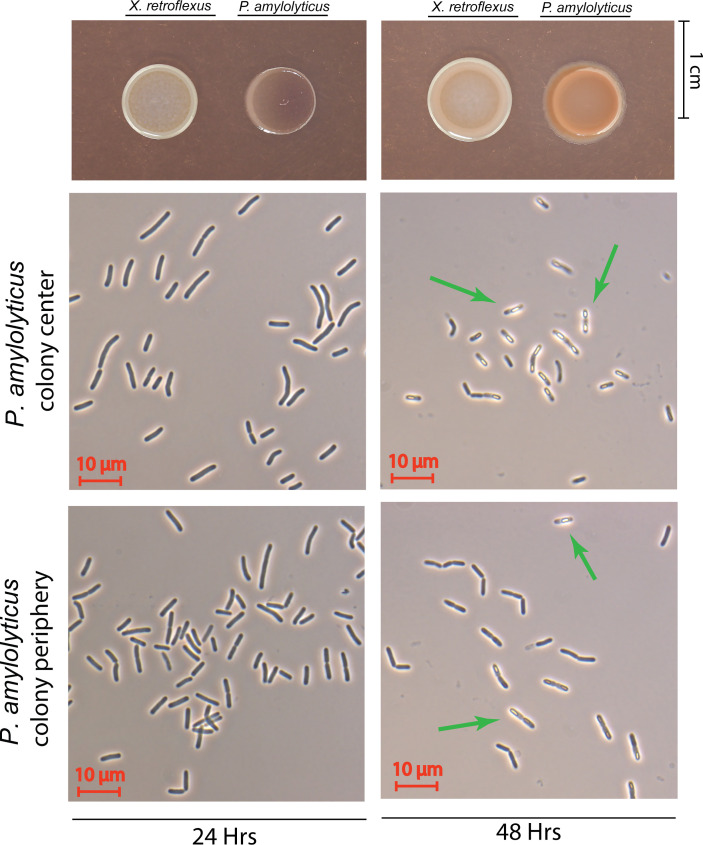
Phase contrast microscopy of the interacting *P. amylolyticus* colony. At 24 hrs neither the colony center nor the colony periphery showed any visual sporulating bacteria. After 48 hrs the presence of sporulating bacteria was observed in the center of the colony, whereas no visible sporulation was observed at the colony periphery. Green arrows mark bacteria with visible spores. Microscopy images of non-interacting *P. amylolyticus* is available as **Figure S15**.

We performed standard phase contrast light microscopy analysis of the interacting and non-interacting *P. amylolyticus* colony. For both the interacting and non-interacting colony of *P. amylolyticus* at 24 hrs, spores were not observed neither at the colony periphery nor centre (**Figures 5** and **S15**). At 48 hrs, spores were observed in both the periphery and centre of the interacting colony (**Figure 5**), whereas no spores could be visually observed in the non-interacting colony (**Figure S15**). At 72 hrs, spore formation was also observed in the centre of the non-interacting colony (**Figure S15**). However, whether this sporulation response was related to a lack of nutrients or a very low pH in the environment is unknown. If the sporulation was related to a lack of nutrients similar to what could cause the sporulation for the interacting colony, then it would be expected that the 72 hrs non-interacting colony would approximate the colony density and morphology of the interacting colony. Hence, it is very likely that the response is due to something else, e.g. a low pH environment.

### Proteomics Characterization of Two-Species Interaction

**Figure 6:**
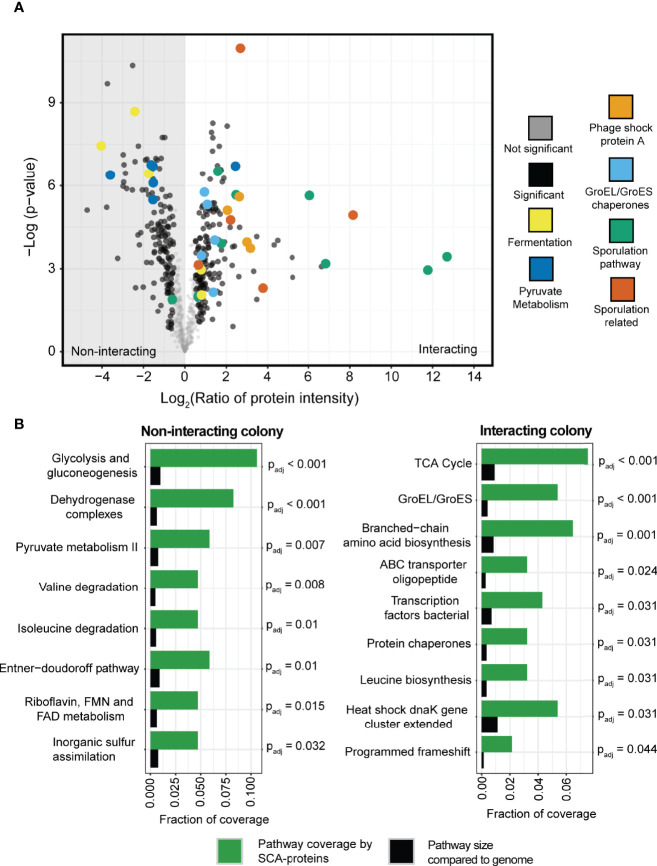
Proteomics profiling of the interacting and non-interacting colony of *P. amylolyticus*. **(A)** Volcano plot of the relative abundance of the proteins between the interacting and the non-interacting *P. amylolyticus* showed a significant change in the abundance of 328 proteins between the two states. **(B)** Identification of potentially regulated pathways between the two conditions. A Fishers Exact test was performed with Benjamini-Hochberg false discovery rate correction (threshold of Padj < 0.05) between pathway coverage with SCA-proteins belonging to the pathway (indicated in green bars) and the proportion of the pathway size in the genome (indicated with black bars).

To further investigate the interacting *P. amylolyticus* colony, as compared to the non-interacting colony, the phenotypic shift was assessed by LC-MS/MS based proteomics profiling. Sample quality overview is appended as **Figure S16** and **S17**. Comparison of interacting and non-interacting *P. amylolyticus* colonies after 48 hrs of growth revealed that many proteins were significantly changed in abundance between the two conditions (**Figure 6**). More than 1100 proteins were identified in both conditions, and of these 328 were significantly changed in relative abundance between the two conditions, referred to as significantly changed in abundance proteins (SCA-proteins). Among the SCA-proteins with the largest change in abundance for the interacting *P. amylolyticus* were proteins directly related to the sporulation pathway and proteins related to sporulation. Proteins included in the group ‘sporulation related’ included: i) a serine protein kinase (PrkA protein) (log2(Ratio) = 8.16) that has been associated with sporulation in *Bacillus subtilis* (Yan et al., 2015), ii) a flotillin 1 (log2(Ratio) = 3.78) that has been associated with sporulation in *Bacillus subtillis* with gene deletion leading to a delay in the onset of sporulation along with reduced sporulation efficiency (Donovan and Bramkamp, 2009; Lopez and Kolter, 2010; Lopez and Koch, 2017), iii) a lipo-protein YhcN precursor that has been related to e.g. the efficiency of spore germination in *Bacillus subtilis* (Bagyan et al., 1998; Watabe et al., 2002; Johnson and Moir, 2017), iv) a YdfA_immunity σ^W^ Regulon which is activated upon damage to the cell envelope such as that caused by spore related cannibalism (Ellermeier, 2006; Ellermeier et al., 2006), antibiotic stress (Cao et al., 2002) or alkaline shock (Schöbel et al., 2004), and v) a competence regulator ComK, YlbF/YmcA, that has been associated to sporulation by accelerating the phosphorylation of Spo0A (Carabetta et al., 2013). Both the YdfA_immunity σ^W^ regulon and the competence regulator ComK, YlbF/YmcA were annotated as hypothetical proteins from the RAST pipeline (Aziz et al., 2008; Overbeek et al., 2014), but were further annotated by PFAM (Finn et al., 2016) annotations using the HMMER service (Potter et al., 2018). This observation strongly supports that active sporulation occurs in the interacting *P. amylolyticus* colony.

The abundance of several proteins related to Phage Shock Protein A was also observed to increase significantly for the interacting colony, which indicates that the cells undergo a stress response related to perturbations of the cell envelope (Flores-Kim and Darwin, 2016), which could be hypothesized to also include pH stress.

To complement the proteomics analysis and further validate the observed proteome changes, a Fisher’s exact test with Benjamini Hochberg FDR correction (Benjamini and Hochberg, 1995) was applied to identify upregulated pathways. For example, the pathways related to the protein chaperones GroEL and GroES as well as the chaperones in the *dnaK* gene cluster were identified as pathways with a significant coverage of SCA-proteins as compared to the size of the pathway in the genome. This further supported that the interacting *P. amylolyticus* was undergoing a stress response. A clear change in metabolic activity was also observed between the interacting and non-interacting colony, based on the identified significantly upregulated pathways. The non-interacting colony had significant coverage of pathways such as glycolysis and gluconeogenesis, the Entner-Duodoroff pathway, pyruvate metabolism and degradation of valine and isoleucine. In contrast the interacting colony had significant coverage of pathways such as branched-chain amino acid biosynthesis and TCA cycle, and ABC oligopeptide transporters.

Comparisons of the proteomes of interacting and non-interacting *X. retroflexus* colonies revealed several SCA-proteins (**Figure S18**). No specific trend could be observed when mapping metabolic functions to the SCA-proteins. To understand the metabolic background for alkalization of the environment, we looked for identified proteins related to amino acid catabolism. Although none of the proteins were significantly changed in abundance when comparing interacting and non-interacting *X. retroflexus* colonies, the glutamate dehydrogenase (EC 1.4.1.2), agmatine deiminase (EC. 3.5.3.12) and N-carbamoylputrescine amidase (EC.3.5.1.53) were observed among identified proteins in both conditions. The glutamate dehydrogenase (EC 1.4.1.2) is involved in oxidative deamination of glutamate to alpha-ketoglutarate and ammonia, and agmatine deiminase (EC. 3.5.3.12) and N-carbamoylputrescine amidase (EC.3.5.1.53) are involved in catabolism of agmatine resulting in generation of ATP and ammonia. The arginine deiminase was not observed in the dataset albeit being present in the genome of *X. retroflexus*. Combined, this indicates a dual approach to amino acids catabolism of *X. retroflexus* and a subsequent release of ammonia into the external environment.

### Summary

**Figure 7:**
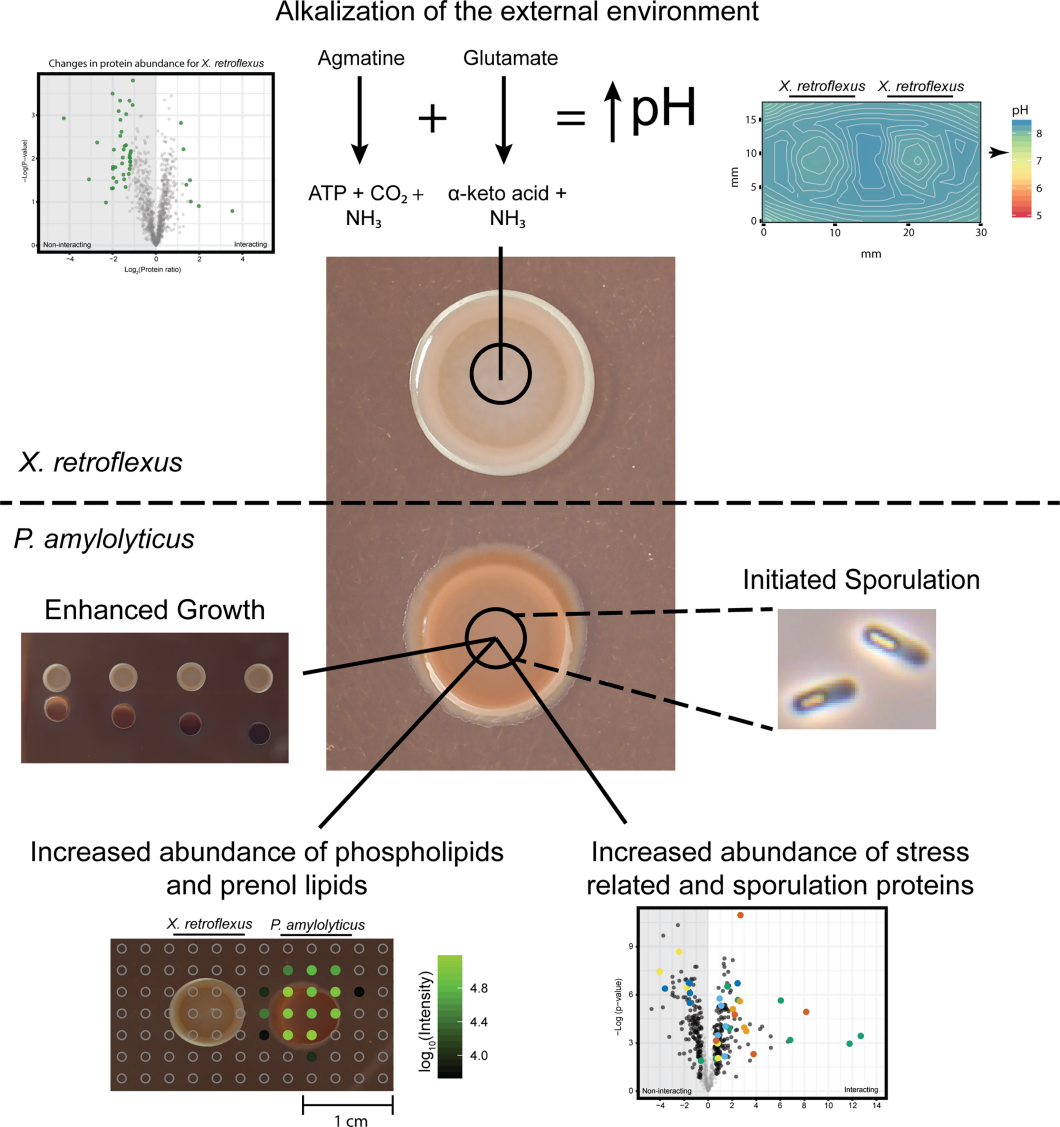
Schematic overview of findings in the study and their relation to the applied techniques. *X. retroflexus* was found to drive environmental pH through degradation of amino acid products. In response, the stabilized pH environment led to; i) enhanced growth of *P. amylolyticus* as identified by increased CFU counts, ii) a changed abundance of glycerophospholipids and prenol lipids in the colony of *P. amylolyticus* as observed from metabolomics, iii) a significantly increased abundance in stress and sporulation related proteins in *P. amylolyticus*, and finally iv) an initiated sporulation response by *P. amylolyticus*, observed by microscopy.

**Figure 7** depicts the specific individual observations from the applied technologies used to unravel the interaction between *X. retroflexus* and *P. amylolyticus*. Application of micro-sensors revealed a strong environmental alkalization by *X. retroflexus*, and subsequent analysis by proteomics indicated this to be driven by degradation of amino acids, e.g. degradation of agmatine and glutamate. In response to the changes in the external environment, *P. amylolyticus* displayed induced growth, and a change in colony morphology, supposedly related to a change in glycerophospholipids and prenol lipids, as indicated by metabolomics. Additionally, proteomics showed a shift towards sporulation, that in part could also contribute to the change in colony morphology, and ongoing sporulation was supported by microscopy images. Notably, after growth for 24 hrs no spores were observed by microscopy in *P. amylolyticus* colonies, but glycerophospholipids and prenol lipids were observed at the part of the *P. amylolyticus* colony facing *X. retroflexus*, where also a minor morphological change was observed. This hints that the environmental alkalization initiates a response of *P. amylolyticus* to manage the changed pH environment by altering its membrane structure and composition, e.g. by altering the chemical composition of its glycerolphospholipids and levels of prenol lipids. In support, σW has been shown to be activated during alkaline shock (Schöbel et al., 2004). Furthermore, our proteomics pathway analysis revealed that protein chaperones, such as GroEL/S system, and proteins encoded by the heat shock *dnaK* gene cluster, were increased in relative abundance during the interaction. Previous studies have shown that sporulation in *Bacillus megaterium* does not increase the levels of GroEL and *dnaK* chaperones (Hlaváček et al., 1998), which indicates a stress response in *P. amylolyticus* potentially related to pH drift.

Here we show how enhanced growth can be facilitated for *P. amylolyticus* through pH stabilization of the environment through metabolic activity of *X. retroflexus*. Indeed, enhanced community growth through facilitated pH stabilization of the local environment is one of the types of inter-species interactions that lacks support from observations in natural systems. Hence, it is not known to which extent this type of interaction plays a role in community development and function, and this needs to be further investigated in future efforts. However, as pH is a strong driver for microbial community composition and functionality, it could be hypothesized that this type of interaction is important to allow a larger local diversity in a community. The present results combined with previous studies (Herschend et al., 2018; Ratzke and Gore, 2018), emphasize pH facilitation as a major factor shaping community ecology.

Here we demonstrate the power of combining high-resolution and high-throughput applications, i.e. microsensors, metabolomics, proteomics and microscopy, to reveal community interactions. Despite the usefulness and application potential of each individual method, the combined interpretation of the individual observations is what eventually provides a convincing identification of the population dynamics and mechanisms underpinning the two-species interaction. Like for microbial communities, each component works well in isolation, but only when parts are combined, synergies arise.

## Materials and Methods

### Cell Cultures

The investigated species included in this study, i.e. *X. retroflexus retroflexus (X. retroflexus)* and *P. amylolyticus amylolyticus (P. amylolyticus)*, were isolated from soil during a previous study on plasmid transfer among soil isolates (de la Cruz-Perera et al., 2013) and were later found to be involved in synergistic biofilm formation (Ren et al., 2015). Bacterial isolates were stored as glycerol stocks at -80°C. Prior to cultivation, the bacterial isolates were streaked on agar plates complemented with tryptic soy broth (TSA) (15 g/L agar and 30 g/L tryptic soy broth, VWR) from the glycerol freeze stocks. Plates were incubated for 48 hrs at 24°C, until single colonies could be selected. Single colonies were used to inoculate 5 mL tryptic soy broth (TSB) (30 g/L tryptic soy broth, VWR) in test tubes and incubated over-night at 24°C with shaking at 250 rpm. Over-night cultures were diluted to an optical density (OD) of 0.15 at 600 nm in TSB before being used in the experimental setups.

### Agar Plates

Bacterial interactions were analyzed on 50% TSA plates (15 g/L agar, Sigma, and 15 g/L tryptic soy broth, Sigma-Aldrich). To allow easier visualization of bacterial interactions 50% TSA plates were complemented with 40 µg/mL Congo red (Fluka) and 20 µg/mL Coomassie brilliant blue G250 (Sigma-Aldrich). Plates complemented with Congo red and Coomassie brilliant blue were only used for photographic purposes and visual inspections. Bacterial cultures diluted to OD=0.15 were spotted onto 50% TSA plates, using 5 µL of the OD adjusted culture. Plates were allowed to dry at room temperature before being incubated at 24°C.

### V-Plates

OD-adjusted bacterial culture (5µL) was spotted on a 50% TSA plates. Square petri dishes, were used for the 50% TSA plates and each plate contained 80 mL 50% TSA. Colonies A-D were cut from the agar plates using a sterile scalpel. Each colony was transferred to a bead-beating tube, 2 mL Free Standing Ribbed Screw Tubes (SSI), with Standard Screw Tube Caps (SSI) containing six 2.5 mm steel beads. Weight of the agar plug was measured. 1 mL 1xPBS was added to each tube and samples were homogenized on a FastPrep^®^-24 (MP), running at speed 4.0 for 3 rounds of 30 sec, with a 1 min resting period between runs. Homogenized samples were 10-fold serial diluted in 1x PBS and 100 µL of the diluted samples were used for CFU counting on TSA plates by plate spreading.

### Micro-Sensor Measurements of pH and Oxygen

2D mapping of the chemical environment was conducted by mounting micro-sensors on a custom-built x-y-z motorized micromanipulator setup fixed to a heavy stand (Lichtenberg et al., 2017). Similar motorized x-y-z micromanipulator setups can be obtained from commercial sources; e.g. *Pyro-Science GmbH, Aachen, Germany* or *Unisense A/S, Aarhus, Denmark.*

A fiber-optic O_2_ microsensor (OXR50-HS, tip diameter 50 µm, Pyro-Science GmbH Aachen, Germany) was used for O_2_ measurements. The sensor was connected to an O_2_ meter (FireStingO2, Pyro-Science GmbH Aachen, Germany). Microsensor calibration was performed as specified by the manufacturer.

Measurements of pH were conducted with a pH glass microelectrode (tip diameter 50 µm, pH50, Unisense A/S) in combination with a reference electrode (tip diameter of ~5 mm, Unisense A/S) immersed in the agar plate. Sensors were connected to a high impedance pH/mV-Meter (Unisense A/S). The pH microelectrode was linearly calibrated from sensor mV readings in pH buffers pH 4, 7 and 9, showing a log-linear response to [H^+^] of ~51 mV/pH unit at experimental temperature (24°C ± 0.5°C).

To control precision of the sensors, sensor movement was recorded with a USB microscope (dino-lite.eu, model AM7515MZTL). 2D measurements (pH and O_2_) were conducted at a depth of ≈100 µm below the surface of the agar plate. A custom-made profiling software (Volfix; programmed by Roland Thar) was used to control the x-y-z motorized micromanipulator and to read out sensor signals. A similar software, Profix, can be downloaded free of charge from pyro-science.com. An analog to digital converter (ADC-101, Pico Technology, UK) had to be used in order to interface the profiling software with the O_2_ meter (using the analog output of the FireStingO2) and the pH/mV-Meter. Time course measurements, of e.g. O_2_, in static culture were recorded with free logging software (SensorTrace logger, Unisense A/S).

### Ammonia Measurements

The ammonia measurements were performed on agar plugs (2.5 by 2.5 mm) excised across the interaction zone. The plugs were incubated in 200 µL MilliQ water for 30 min and then centrifuged at 4000 *g* for 10 min. Subsequently the ammonia concentration was measured with Ammonia Assay Kit (Sigma-Aldrich) according to manufacturers instructions. For each agar plate a reference measurement was acquired by extracting a single 2.5 by 2.5 mm agar plug near the agar plate periphery. Ammonia concentrations in agar plugs transecting the interaction zone were normalized to this value, and the average change in ammonia was calculated across three biological replicates.

### Metabolomics

#### Metabolite Extractions

OD 0.15 adjusted bacterial cultures of *X. retroflexus* and *P. amylolyticus* were used. 5 µL cell cultures were spotted onto 50% TSA plates, according to the plate setups presented in e.g. **Figure S6**, producing agar plates with either *X. retroflexus- X. retroflexus*, *P. amylolyticus-P. amylolyticus* or *X. retroflexus-P. amylolyticus* interactions. Three technical replicates were made of each plate for each sampling time point. 2.5 by 2.5 mm agar plugs were excised from each technical replicate plate and the agar plugs were transferred to methanol pre-washed 96 well plates (Thermo Scientific, Nunc™ 96-Well Polypropylene Storage Microplates). For each type of interaction zone (e.g. *X. retroflexus-P. amylolyticus*) agar plugs from one technical replicate were extracted with 150 µL methanol. Another technical replicate was extracted with 150 µL butanol, and the third replicate was extracted with 150 µL ethyl-acetate. Solvent was added and 96 well plates were sealed with 96 well-cap mats (Thermo Scientific, Nunc 96 well-cap mats) and incubated for 60 min with slow horizontal shaking. After incubation, 100 µL was transferred from each solvent extraction and the three solvent extractions were pooled together for each excised spot in a methanol pre-washed 96 well plate. Solvent was evaporated in a SpeedVac and the resulting pellet was re-dissolved in 20% methanol for 1 hrs at slow horizontal shaking. Samples were centrifuged at 5000 *g* for 10 min to pellet potential undissolved material and the supernatant was transferred to a new methanol pre-washed 96 well plates which was sealed with Zone-Free Sealing Film (Excel Scientific) prior to analysis by liquid chromatography coupled tandem mass spectrometry.

#### Liquid Chromatography Coupled Mass Spectrometry Analysis for Metabolomics

MS analysis was performed on a micrOTOF-Q II (Bruker Daltonics) mass spectrometer with an electrospray ionization (ESI) source, controlled by OTOF control and Hystar. MS spectra were acquired in positive ion mode over a mass range of 100–2,000 *m/z*. An external calibration with ESI-L Low Concentration Tuning Mix (Agilent Technologies) was performed prior to data acquisition and on every day during acquisition. Hexakis(2,2-difluoroethoxy)phosphazene (*m/z* 621.19, Sigma-Aldrich) was used as a lock mass internal calibrant during data acquisition. The following instrument settings were used for the MS data acquisition: capillary voltage of 4,500 V, nebulizer gas (nitrogen) pressure of 3 bar, ion source temperature of 200°C, dry gas flow of 9 l min^–1^, source temperature and spectra acquisition rate of 3 Hz for MS1 and MS2. A Top10 method was applied to select MS1 ions for fragmentation per MS1 scan. Fragmentation was performed by collision-induced dissociation (CID) according to the following fragmentation and isolation list (values are *m/z*, isolation width and collision energy, respectively): 100, 4, 16; 300, 5, 24; 500, 6, 30; 1,000, 8, 40; 1,500, 10, 50; 2,000, 12, 70. In addition, the basic stepping function was used to fragment ions at 100 and 160% of the collision-induced dissociation (CID) energy calculated for each *m/z* from the above fragmentation and isolation list with a timing of 50% for each step. The basic stepping had a collision radiofrequency (RF) of 198 and 480 peak-to-peak voltage, Vpp, with a timing of 50% for each step and transfer time stepping of 75 and 92 μs with a timing of 50% for each step. The MS/MS active exclusion parameter was set to 5 and released after 0.5 min. The injected samples were chromatographically separated using an Agilent 1290 Infinity Binary LC System (Agilent Technologies) controlled by Hystar software (Bruker Daltonics), using a 50 × 2.1 mm Kinetex 1.7 μM, C18, 100 Å chromatography column (Phenomenex), 30°C column temperature, 0.5 ml min^-1^ flow rate, mobile phase A 99.9% water (J.T.Baker, LC-MS grade) with 0.1% formic acid (Fisher Scientific, Optima LC/MS), mobile phase B 99.9% acetonitrile (J.T.Baker, LC-MS grade) with 0.1% formic acid (Fisher Scientific, Optima LC/MS), with the following gradient: 0–0.5 min 10% B, 0.5–1 min 50% B, 1–6 min 100% B, 6–9 min 100% B, 9–9.5 min 10% B, 9.5–10 min 10% B.

Blank injections, without liquid, were performed for every 12th sample along to check for potential column carry over. Blank agar control were submitted after 3 blank injections.

### Metabolomics Data Analysis

LC-MS/MS data were converted to the.mzXML data format and lock mass correction was performed using Compass Data Analysis (Bruker Daltonics). Subsequently, .mzXML files were preprocessed using MZmine 2.3 (Pluskal et al., 2010) with parameters set to: Peak detection/Mass detection/Mass detector, Centroid, MS1 noise level 2.0E2, MS2 noise level 0, Chromatogram builder/MS1 level, Min time span (min) 0.05, Min height 6.0E2, *m/z* tolerance 20 ppm, Chromatogram deconvolution/Algorithm, Local minimum search, *m/z* range for MS2 scan pairing (Da) 0.025, RT range for MS2 scan pairing (min) 0.1, Chromatogram threshold 30%, Search minimum in RT range (min) 0.10, Minimum relative height 5%, Minimum absolute height 8.0E2, Min ratio of peak top/edge 1, Peak duration range (min) 0.05-2, Isotopic peak grouper, *m/z* tolerance 20 ppm, Retention time tolerance 0.1 (min), Maximum charge 3, Representative isotope Most intense, Feature alignment, *m/z* tolerance 20 ppm, Weight for *m/z* 50, Retention time tolerance 0.1, Weight for RT 50. An MS2 filtered.mgf file was then exported from MZmine and uploaded to the Global Natural Products Social Molecular Networking webserver (http://gnps.ucsd.edu) for mass spectral molecular networking using the following settings: Precursor Ion Mass Tolerance 0.02 Da, Fragment Ion Mass Tolerance 0.02 Da, Min Pairs Cos 0.65, Min Matched Fragment Ions 5, Network TopK 10, Minimum Cluster Size 1, Maximum Connected Component Size 100, Run MS Cluster no. Mass spectral molecular networks visualize MS2 spectral similarity in the form of a network. Each node in the network represents a mass spectral molecular feature and nodes connected with an edge represent MS2 spectra with high spectral similarity, assessed through a cosine similarity score (Watrous et al., 2012). High similarity of MS2 fragmentation spectra can be used as a proxy for chemical structural similarity. Thus, connected nodes likely represent chemically structurally similar molecules. To retrieve relative abundances per mass spectral molecular feature, an MS2 filtered feature table consisting of integrated peak areas was exported from MZmine and an in-house script in python was used to calculate mean peak areas per feature and per sample group (agar blank, dual-species interaction zone, *X. retroflexus* and *P. amylolyticus*). The mass spectral molecular network with mapped mean peak areas per sample group was visualized using Cytoscape version 3.4.0 (Shannon et al., 2003). The mass spectral molecular network job is publicly available at (https://gnps.ucsd.edu/ProteoSAFe/status.jsp?task=7dd2927952f14278b3aaa6e113833bd8) and.mzXML files were uploaded to MassIVE (https://massive.ucsd.edu) and are publicly available under the accession no. MSV000082481. The script used to calculate mean peak areas per sample group is publicly available as a Jupyter notebook at (https://github.com/madeleineernst/MetProfiling_Pamylolyticus_vs_Xretroflexus). The described workflow corresponds to a prototype version of the feature-based molecular networking workflow (Nothias et al., 2020). The mass spectral molecular network consisted of 5135 nodes, organized in 198 independent molecular families (two or more connected components of a graph) and a total of 3320 single nodes with no spectral similarity to any other node. **Figure S6A** shows an overview of the global mass spectral molecular network with nodes colored according to the sample group in which the mass spectral molecular feature was detected (agar blank, dual-species interaction zone, *X. retroflexus* and *P. amylolyticus*).

To putatively identify mass spectral molecular features within our network, we used GNPS spectral library matching, which resulted in a putative annotation rate of approx. 2% of the nodes. This annotation rate is not atypical as many molecules, particularly of microbial origin, are unique and not contained in reference spectral libraries (da Silva et al., 2015). To further enhance putative structure annotation we performed *in silico* structure annotation through Network Annotation Propagation (NAP) (da Silva et al., 2018) with parameters set to 10 first candidates for consensus score, fusion result for consensus enabled, 30 ppm accuracy for exact mass candidate search, cosine value to subselect inside a cluster 0.65, adduct ion type [M+H] and [M+Na], maximum 10 candidate structures in the graph, skip parent mass disabled and the following structure databases: GNPS, HMDB, SUPNAT, CHEBI, FooDB. NAP results are publicly accessible at: (https://proteomics2.ucsd.edu/ProteoSAFe/status.jsp?task=d1ad37ab1e054609bf888d4aa85edf4f and https://proteomics2.ucsd.edu/ProteoSAFe/status.jsp?task=0b369206f6a64f33a6704f3286824da1). Additionally, to enhance *in silico* putative structure annotation of peptidic molecules we submitted the same .mgf file used for mass spectral molecular networking through GNPS to Dereplicator (Mohimani et al., 2017), both with and without analog search and with parameters set to Percursor Ion Mass Tolerance 0.02 Da, Fragment Ion Mass Tolerance 0.02 Da, PNP database Extended, Max Charge 2, Accurate P-values enabled, Min Number of AA 3, Max Isotopic Shift 2, Adducts Na and K, Max Allowed Modification Mass 150 Da, Min Matched Peaks with Known Compound 4. Dereplicator results are publicly accessible at (https://gnps.ucsd.edu/ProteoSAFe/status.jsp?task=f885fda8e67c40968aa3896b6c7a042a and https://gnps.ucsd.edu/ProteoSAFe/status.jsp?task=d3354afc0ddc479ba7ab43630 45b352c).

Finally, to get a more easily interpretable overview of putative structure identification we submitted all structures retrieved *in silico* or through GNPS library matching to automated chemical classification using ClassyFire (Feunang et al., 2016) and calculated most predominant chemical classes per mass spectral molecular family (two or more connected components of a graph) at each hierarchical level of the ClassyFire ontology using MolNetEnhancer (Ernst et al., 2019). A score was calculated representing the degree of predominance of a chemical class within a molecular family. A MolNetEnhancer score of 1 corresponds to a scenario, where all predicted structures fall within the same chemical class category, whereas a score close to 0 corresponds to a very low degree of consensus of the predicted structures, more likely resulting from false positive predictions. The script used to retrieve ClassyFire chemical classes per mass spectral molecular family is publicly available as a Jupyter notebook at (https://github.com/madeleineernst/MetProfiling_Pamylolyticus_vs_Xretroflexus).

This resulted in putative structures or suggestions for chemical classes retrieved for over 1800 nodes within the network (35%) corresponding to a level 2 or 3 of metabolite identification according to the Metabolomics Standard Initiative’s reporting standards (Sumner et al., 2007).

#### Spatial Visualization of Metabolomics Data

Metabolite distribution were screened using the ‘ili software (Protsyuk et al., 2017) by mapping MS1 intensities of detected features onto 2D pictures of the respective interaction zone. Final plotting of metabolite distributions for the manuscript was performed in the R environment (R Core Team, 2018) using ggplot2 (Wickham, 2009) and grid (Murrell, 2011). MZmine preprocessing resulted in peak splitting in some cases, which however could be easily identified when assessing the spatial distribution of the mass spectral molecular features. Feature peak areas were summed in these cases.

#### Comparison of Ion Intensities Across Interacting and Non-Interacting Colonies

For interacting and non-interacting *P. amylolyticus*, 12 spots covering the inner part of the colony were selected. MS1 ion intensities for the interacting colony were divided by 3 to account the approximate 2.5-fold higher cell counts observed in the interacting colony. For blank spots on the interacting and non-interacting colony, a threshold MS1 ion intensity equal to the lower 1% quantile of detected MS1 ion intensities was inferred. The intensity ratio across spots was then compared across the colony by a linear model with offset = 0, followed by FDR correction.

### Proteomics

#### Protein Extraction

All proteomics experiments were conducted on 50% TSA plates without Congo red and Coomassie brilliant blue. Colony biomass was extracted from the 50% TSA plates using a scalpel. *X. retroflexus* cell biomass was dissolved in protein extraction buffer (200 mM Tris HCl pH 7.6, 200 mM NaCl, 1 mM EDTA, 5% glycerol and 20 mM DTT) and the cells were lysed with a Q500 sonicator (Qsonica, Newtown, USA) for 4 min at 30% amplitude. The sonicated samples were centrifuged at 10000 g and soluble proteins in the supernatants were precipitated by adding ice-cold acetone to a final concentration of 80%. Samples were precipitated over night at -18°C. Precipitated proteins were resuspended in a urea solution (8M urea, 50 mM ammonium bicarbonate, pH 8). For *P. amylolyticus* the samples were dissolved in 8M guanidine hydrochloride and sonicated with similar parameters as *X. retroflexus*. Protein concentration was estimated *via* Bradford assay and samples were diluted to 5 μg/μL, reduced with 5 mM DTT for 1 hr, and alkylated with 20 mM iodoacetamide for 1 hr in the dark. Samples were four fold diluted in 50 mM ammonium bicarbonate pH 8, and then incubated overnight by trypsin (1:100 trypsin-to-protein ratio wt:wt) at room temperature with horizontal shaking at 500 rpm. Inactivation of trypsin was achieved by adding trifluoroacetic acid (TFA) to 2% and debris was removed by centrifugation (10000 *g*, 10 min).

The tryptic peptides were purified on STAGE-tip micro columns (Kulak et al., 2014) before being subjected to analysis by LC-MS/MS.

#### Liquid Chromatography Coupled Mass Spectrometry Approach for Proteomics

The samples were analyzed by liquid chromatography tandem mass spectrometry (LC-MS/MS) and data were recorded in a data dependent manner, automatically switching between MS and MS/MS acquisition, on a Q-Exactive (Thermo Scientific, Bremen, Germany). An EASY nLC-1000 liquid chromatography system (Thermo Scientific, Odense, Denmark) was coupled to the mass spectrometer through an EASY spray source and peptide separation was performed on 50 cm EASY-spray columns (Thermo Scientific) with 2 µm size C18 particles and inner diameter of 75 µm. Mobile phase consisted of solvents A (0.1% formic acid) and B (80% acetonitrile in 0.1% formic acid). The initial concentration of solvent B was 6%, and hereafter gradients were applied to reach the following concentrations: 14% solvent B in 37 min, 25% solvent B in 42 min, 38% solvent B in 21 min, 60% solvent B in 20 min, 95% solvent B in 3 min and 95% solvent B for 7 min. The total length of the gradient was 130 min. The full scans were acquired in the Orbitrap with a resolution of 70,000 and a maximum injection time of 20 ms was applied. For the full scans, the range was adjusted to 350-1500 *m/z*. The top 10 most abundant ions from the full scan were sequentially selected for fragmentation with an isolation window of 1.6 *m/z* (Kelstrup et al., 2012), and excluded from re-selection for a 30 s time period. For the MS/MS scans the resolution was adjusted to 17,500 and maximum injection time of 60 ms. The ions were fragmented in a higher-energy collision dissociation (HCD) cell with normalized collision energy (NCE) of 28%.

#### Data Analysis for Raw MS Files

The acquired raw data was analyzed using MaxQuant version 1.5.5.1 (Cox and Mann, 2008) with the inbuilt Andromeda search engine (Cox et al., 2011). Mass tolerance was set to 4.5 ppm (parent ions) and 20 ppm (fragment ions); a maximum of 2 missed tryptic cleavages were permitted. Methionine oxidation and protein N-terminal acetylation were selected as variable modifications. Carbamidomethylation of cysteines was selected as a fixed modification. A target decoy search that allowed for a maximum of 1% FDR on both peptide and protein level and a minimum length of 7 amino acids per peptide was performed. Quantification was performed using the label free quantification (LFQ) algorithm (Cox et al., 2014) in MaxQuant with a minimum ratio count of 2 and applying the match between runs function.

#### Analysis of Identified Proteins

Data analysis was in part performed with Perseus v1.5.3.2 (Tyanova et al., 2016). Proteins with a significant change in relative abundance were identified using a modified Welsh t-test with an S0 parameter of 1 (Tusher et al., 2001), a permutation based FDR cut-off of 0.05 and valid values in at least 60% of the samples in both single and multispecies conditions. Pathway analysis was performed with a Fishers exact test corrected for multiple hypothesis testing with Benjamini Hochberg FDR correction with a threshold for the adjusted p-value of <0.05, in R. All plots were prepared in R v3.3.0 using ggplot2 v2.1.0 (Wickham, 2009).

#### Data Availability of Proteomics Data

Proteomes for analysis in MaxQuant were prepared as part of an earlier investigation; PRJEB18431 (*X. retroflexus retroflexus*) and PRJEB15262 (*P. amylolyticus amylolyticus*). Proteomes were based on the genomes which were annotated with the RAST database (Aziz et al., 2008; Overbeek et al., 2014). Raw files and output from MaxQuant are available *via* ProteomeXchange with identifier PXD031026 for *X. retroflexus* samples and with PXD030998 for *P. amylolyticus*.

## Data Availability Statement

The datasets presented in this study can be found in online repositories. The names of the repository/repositories and accession number(s) can be found below: ProteomeXchange: PXD031026; PXD030998.

## Author Contributions

JH contributed with conception, design, experimental work, statistical analysis, and wrote the first draft of the manuscript. ME performed data management, statistical analysis and wrote sections of the manuscript. KK contributed to study design, experimental work and wrote sections of the manuscript. AM contributed with experimental support for metabolomics, data management and statistical analysis. RS performed data processing and statistical analysis. HR and ZD performed experimental work. PH and BS contributed with support for proteomics and study design. All authors contributed to manuscript revision, read, and approved the submitted version. PD contributed with conception and design of the study. MB contributed with conception and design of the study and wrote sections of the manuscript. All authors contributed to manuscript revision, read, and approved the submitted version.

## Funding

This study was supported by grants from the Independent Research Fund Denmark | Technical and Production Sciences (MK; DFF-8022-00301B, MB; DFF – 1335-00071), Villum Foundation (MB; 10098) and Novo Nordic Foundation (MB; 27620). The Q-Exactive Orbitrap mass spectrometers used in this study were granted by the Independent Research Fund Denmark | Natural Sciences (11-106246) and the Velux Foundation. The Danish Research Foundation, Nordic Bioscience A/S and Technical University of Denmark are acknowledged for a joint PhD scholarship to ZD.

## Conflict of Interest

The authors declare that the research was conducted in the absence of any commercial or financial relationships that could be construed as a potential conflict of interest.

## Publisher’s Note

All claims expressed in this article are solely those of the authors and do not necessarily represent those of their affiliated organizations, or those of the publisher, the editors and the reviewers. Any product that may be evaluated in this article, or claim that may be made by its manufacturer, is not guaranteed or endorsed by the publisher.
