# Supplementary material for: Metabolic Profiling of Interspecies Interactions During Sessile Bacterial Cultivation Reveals Growth and Sporulation Induction in Paenibacillus amylolyticus in Response to Xanthomonas retroflexus
Source: Front Cell Infect Microbiol. 2022 Mar 29;12:805473. doi: 10.3389/fcimb.2022.805473 (PMC9001975; doi:10.3389/fcimb.2022.805473)
Supplement: Supplementary file 1 [file DataSheet_1.pdf]

Metabolic profiling of interspecies interactions during sessile bacterial cultivation reveals growth and sporulation induction in *Paenibacillus amylolyticus* in response to *Xanthomonas retroflexus*

Authors

Jakob Herschend[1], Madeleine Ernst[2,3], Klaus Koren[4], Alexey Melnik[2], Ricardo R. da Silva[2], Henriette L. Røder[1], Zacharias B. V. Damholt[5], Per Hägglund[5,7], Birte Svensson[5], Søren J. Sørensen[1], Michael Kühl[6], Pieter C. Dorrestein[2] and Mette Burmølle[1*]

Affiliations

[1]Section of Microbiology, Department of Biology, University of Copenhagen, Denmark

[2]Collaborative Mass Spectrometry Innovation Center, Skaggs School of Pharmacy and Pharmaceutical Sciences, University of California San Diego, La Jolla, CA, USA

[3]Section for Clinical Mass Spectrometry, Danish Center for Neonatal Screening, Department of Congenital Disorders, Statens Serum Institut, Copenhagen, Denmark

[4]Aarhus University Centre for Water Technology (WATEC), Department of Biology, Section for Microbiology, Aarhus University, Denmark

[5]Department of Biotechnology and Biomedicine, Technical University of Denmark, Denmark

[6]Marine Biological Section, Department of Biology, University of Copenhagen, Denmark

[7]Present address: Department of Biomedical Sciences, University of Copenhagen, Nørregade 10, Copenhagen, DK-1017 Denmark

* Corresponding Author: Mette Burmølle, burmolle@bio.ku.dk

Supplementary tables:

Counts of nodes

Grouping	Nodes in total	Nodes in molecular families	Single nodes
Agar related	3629	1324	2305
Dual-species	84	24	60
Shared single-dual	1075	383	692
Single-species	347	84	263
Total	5135	1815	3320

Table S1: Counts of nodes from GNPS mass spectral molecular networking

Total nodes = 5135
Percentage in clusters = 35.35 %
Percentage that are self-loops = 64.65 %

Counts of molecular families and their associated nodes

Grouping	Counts of molecular families	Counts of molecular families containing multiple nodes	Counts of molecular families containing a single node
Agar related	2491	186	2305
Dual-species	61	1	60
Shared single-dual	712	10	692
Single-species	264	1	263
Total	3528	198	3320

Table S2: Counts of clusters from the GNPS molecular networking

Counts of identifications

Grouping	Nodes with putative chemical class information retrieved	Nodes with GNPS Library Match
Agar related	1324	71
Dual-species	24	1
Shared single-dual	383	20
Single-species	84	14
Total	1817	106

Table S3: Counts of nodes from the GNPS molecular network, which carries putative chemical class information retrieved through the MolNetEnhancer workflow.

Component index	Shared with Single species	*m/z* groups and number of nodes[a]	Fusion SMILE and highest Score	Concensus SMILE and Score	MolNetEnhancer: Class and Score	MolNetEnhancer: Direct Parent and Score	Relevant spatial distribution
70	Yes (X. retro)	m/z 513.496 (1)<br>m/z 513.484 (1)	No	1	Glycerolipids (0.667)	1-monoacylglycerols (0.667)	Yes (in X.retro colony)
90	Yes (X. retro and P.amy)	m/z 211.097 (1)<br>m/z 211.094 (1)<br>m/z211.095 (1)<br>m/z211.103 (1)	No	1	Phenols (0.216)	Methoxyphenols (0.205)	No
93	Yes (X. retro and P.amy)	m/z 282.286 (1)<br>m/z 282.144 (1)<br>m/z 282.15 (1)	No	1	Carboxylic acids and derivatives (0.226)	Morpholines (0.111)	No
113	Yes (P.amy)	m/z 766.543 (1)<br>m/z 752.529 (1)<br>m/z 778.546 (1)	No	1	Glycerophospholipids (1.0)	Monomethylphosphatidylethanolamines (0.35 [illegible]	Yes
114	No	m/z 744.56x (2)<br>m/z 462.302 (1)	No	1	Glycerophospholipids (0.68)	Dimethylphosphatidylethanolami[illegible]	Yes
159	No	m/z 593.3xx (2)<br>m/z 607.379 (3)	No	Yes (score = 1)	Prenol lipids (0.348)	Sterodial Glycoside (0.119)	Yes
168	Yes (X. retro)	m/z 573.299 (1)<br>m/z 573.286 (1)	No	1	Steroids and steroid derivatives (0.29)	Gluco/mineralocorticoids, progestogins and derivatives [illegible]	Yes (in X.retro colony)
218	Yes (X. retro and P.amy)	m/z 449.235 (1)<br>m/z 520.285 (1)<br>m/z 520.26 (1)<br>m/z 562.309 (1)	No	1	Macrolactams (0.24)	Macrolactams (0.24)	No
277	Yes (X. retro)	m/z 580.285 (1)<br>m/z 580.271 (1)	No	Yes (score = 1)	Benzene and substituted derivatives (0.263)	N-phenylureas (0.131)	Yes (in X.retro colony)
347	Yes (X. retro)	m/z 781.539 (1)<br>m/z 781.539 (1)	No	Yes (score = 1)	Prenol lipids (0.667)	Sesquiterpenoids (0.44)	Yes

Table S4: Molecular families with putative *in silico* structure identifications uniquely found within the dual-species interaction zone or in combination with a single-species.

[a] the (x) refers to the number of nodes with the given or closely related *m/z*

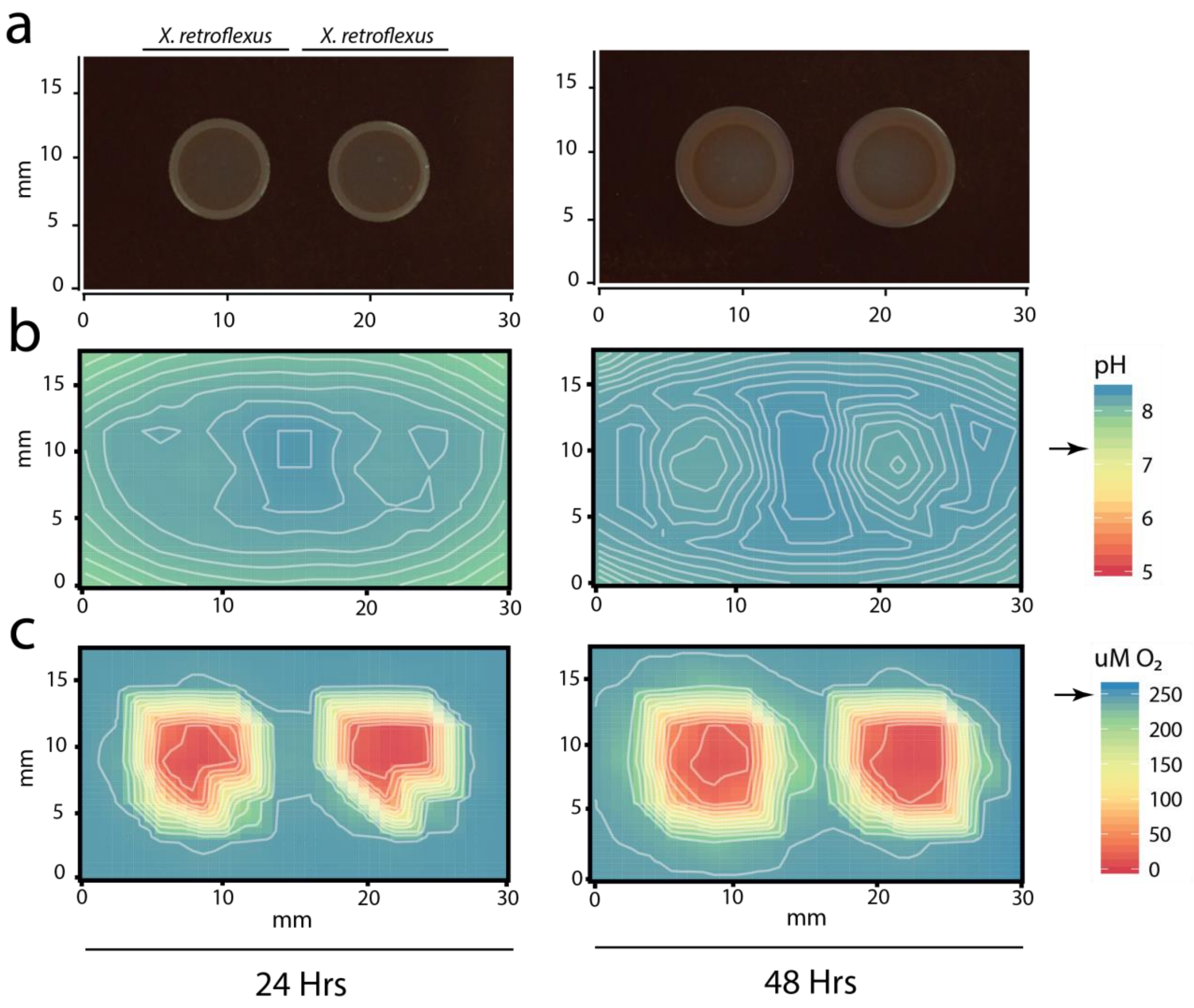

Supplementary Figure S1:

a) Morphology of *X. retroflexus* on TSA. b) Mapping of pH across the interaction zone. Black arrow on scale bar indicates pH of TSA. c) mapping of O_2 concentration around two interacting *X. retroflexus* colonies. Black arrow on scale bar indicates oxygen level in TSA without inoculation of bacteria. When growing alone, *X. retroflexus* drives the pH towards 8.5 after 24 hrs. Towards 48 hrs, the entire environment around the two colonies had increased to pH 8.5. Oxygen profiles measured both at 24 and 48 hrs showed strong O_2 consumption by *X. retroflexus* inducing anoxia in the middle of the colonies.

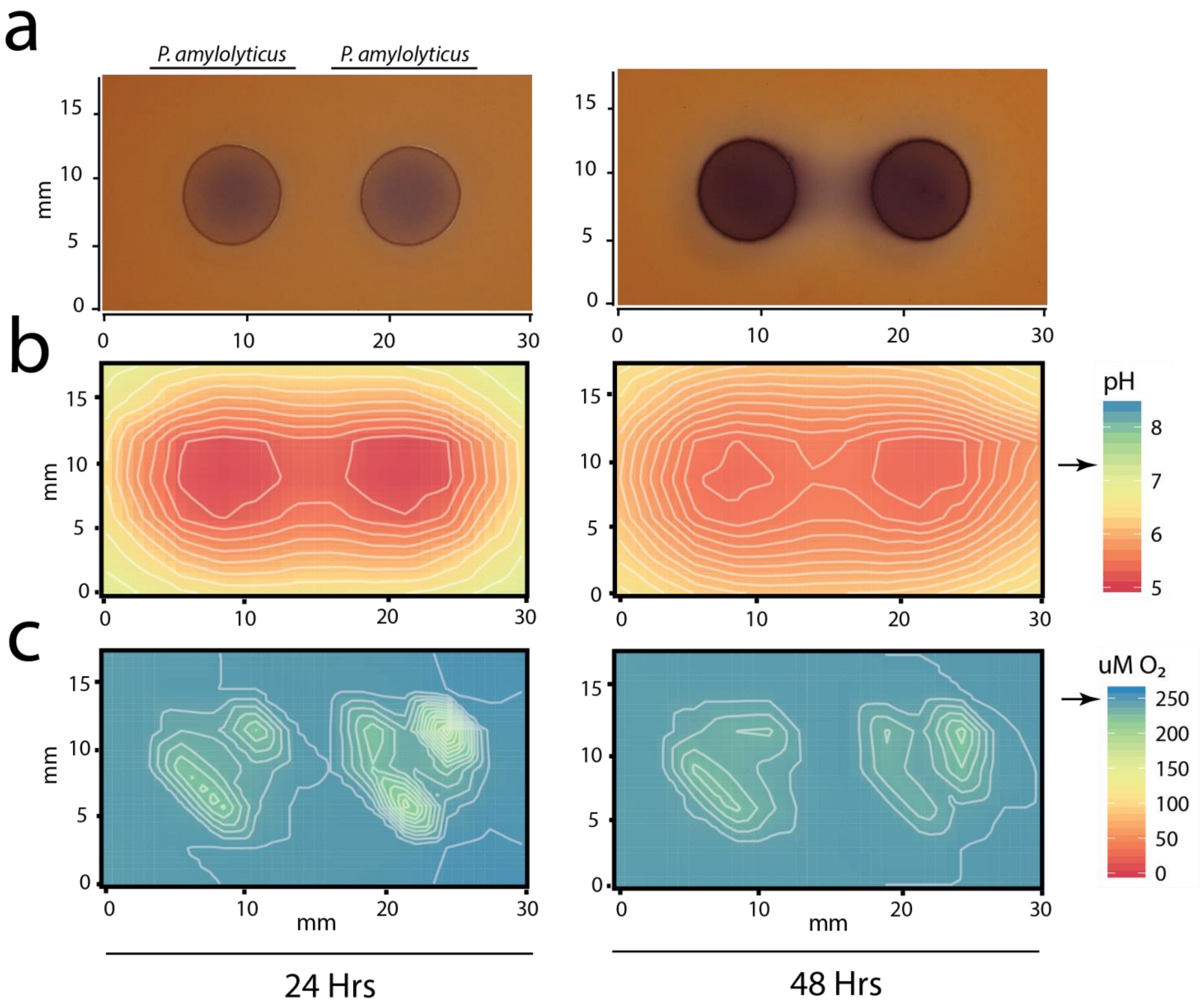

Supplementary Figure S2:

Mapping of pH and O_2 concentration around two colonies of interacting *P. amylolyticus*.

a) Typical morphology of *P. amylolyticus* at 24 and 48 hrs of incubation. b) *P. amylolyticus* changed the pH of the environment to pH 5 at 24 hrs. Towards 48 hrs, the pH in the colonies increased slightly, possibly due to reduced growth and diffusion of buffer compounds in the media. Black arrow on scale bar indicates pH of TSA. c) Oxygen profiles measured at both 24 and 48 hrs indicated that *P. amylolyticus* was mainly respiring in the perimeter of the colony. The overall low O_2 consumption suggest that the majority of the growth is based on fermentation. Black arrow on scale bar indicates oxygen level in TSA without inoculation of bacteria.

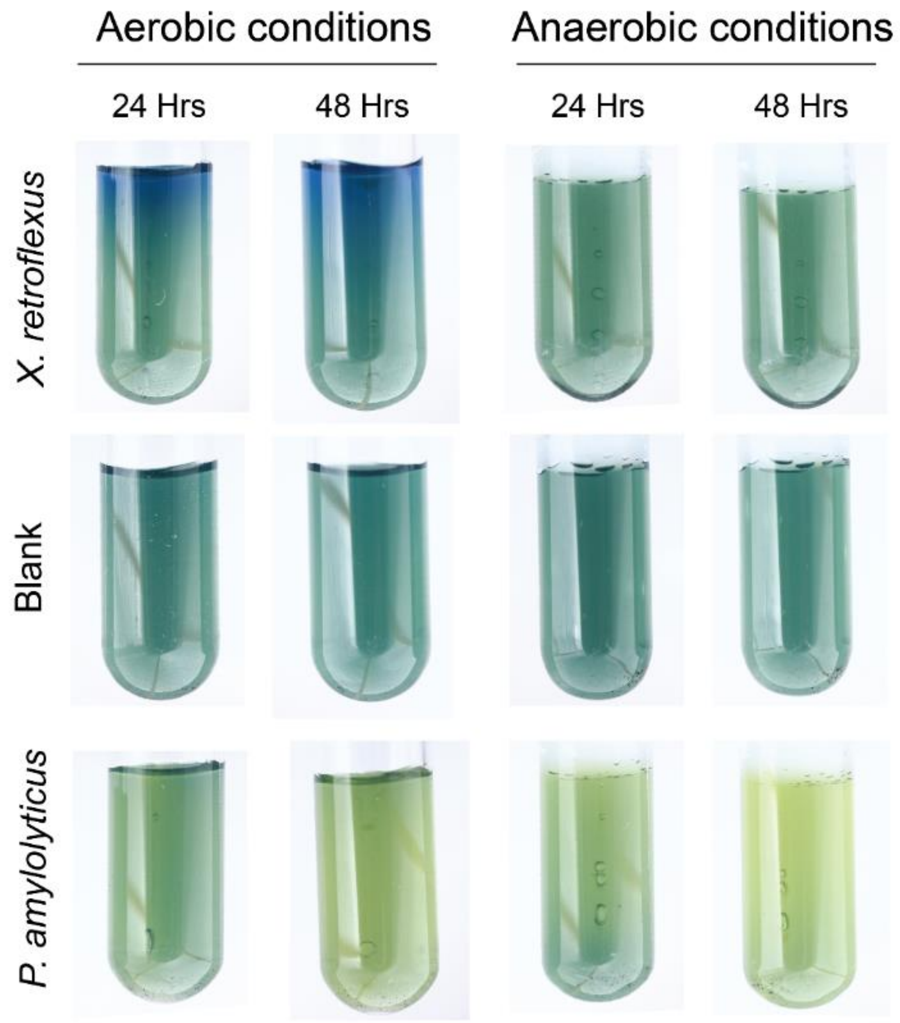

Supplementary Figure S3:
Hugh & Leifson test on individual species at 24 and 48 hrs of growth after inoculation in aerobic and anaerobic vials. Hugh and Leifson media was prepared accordingly; 2 g/L peptone, 5 g/L sodium chloride, 0.3 g/L di-potassium phosphate, 0.03 g/L bromothymol blue, 3 g/L glucose and 3 g/L Agar. Ingredients, except agar, were mixed and pH is adjusted to 7.2. Agar was added and the solution was autoclaved at 121°C for 20 min. Media was poured into test tubes and allowed to solidify. After the media had solidified, the individual species were inoculated into the media from a liquid TSB culture using an inoculation loop. Anaerobic tubes were sealed with melted vaseline immediately after inoculation. Test tubes were incubated for 48 hrs at 24°C. At 24 and 48 hrs of incubation, photos of the tubes were taken with a Canon EOS 700D camera equipped with a Canon EFS 60 mm lense. No color adjustments were made to the photos. Growth of *X. retroflexus* in the aerobic tubes turned the media slightly blue due to alkalization of the medium. No growth was observed in anaerobic tubes inoculated with *X. retroflexus*. Growth was observed in both aerobic and anaerobic tubes with *P. amylolyticus* and yellow media coloration, due to media acidification, was observed. The figure was adapted from Herschend et al. 2018.

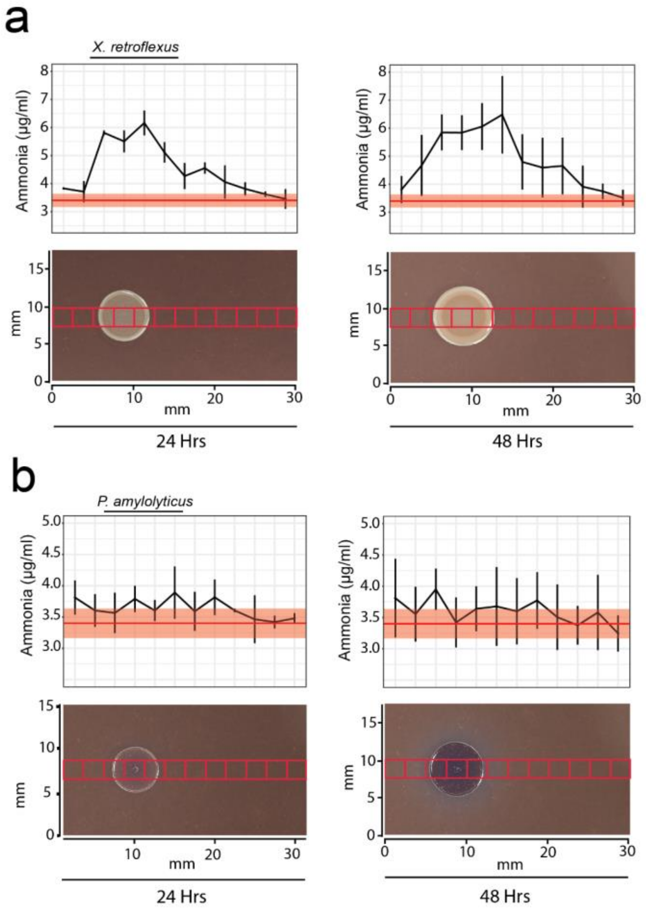

Supplementary Figure S4:

Ammonia concentration profile measured across non-interacting colonies of *X. retroflexus* and *P. amylolyticus*. 2.5 x 2.5 mm agar plugs were excised across the TSA plates and the amount of ammonia was measured in each agar plug. Red line indicates average ammonia concentration in blank agar plugs (n=3) (50% TSA plates). Black line represents mean ammonia concentration across biological replicates (n=3). At 24 hrs ammonia was released from *X. retroflexus* and an ammonia gradient formed across TSA plates. At 48 hrs the amount of ammonia was more dispersed across the TSA plates. The *P. amylolyticus* colony exhibited limited or no production of ammonia at 24 hrs and this did not substantially change after 48 hrs. All error bars are standard error of means.

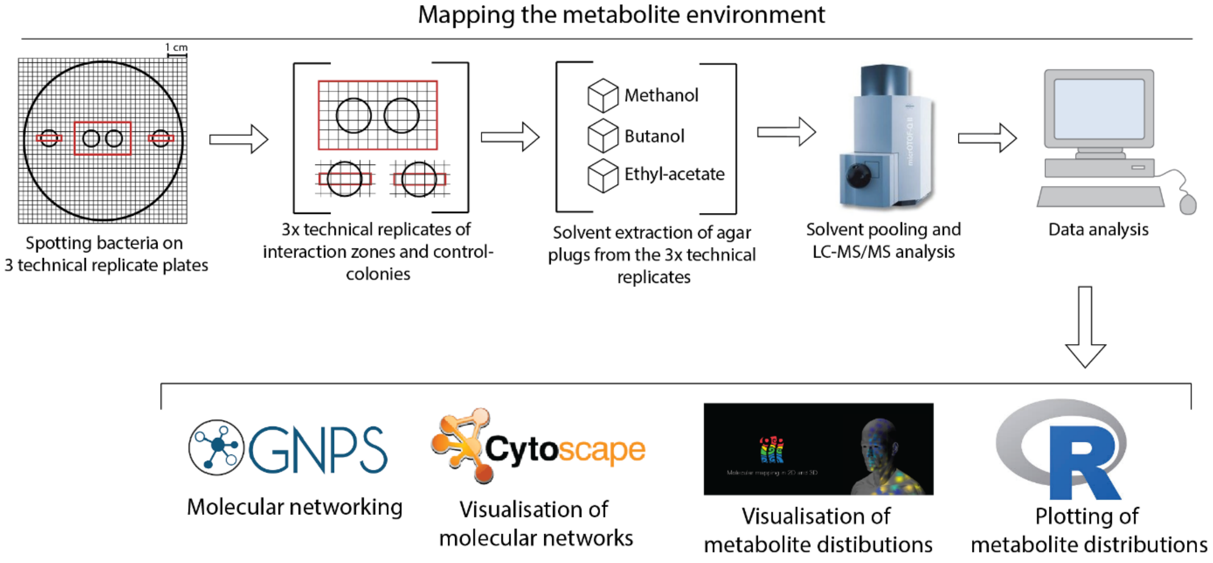

Supplementary Figure S5:
Schematic overview of the metabolite extraction and identification from bacterial interaction zones. Agar plugs were cut from the agar plate in 2.5 x 2.5 mm square plugs across the interaction zone according to the presented scheme.

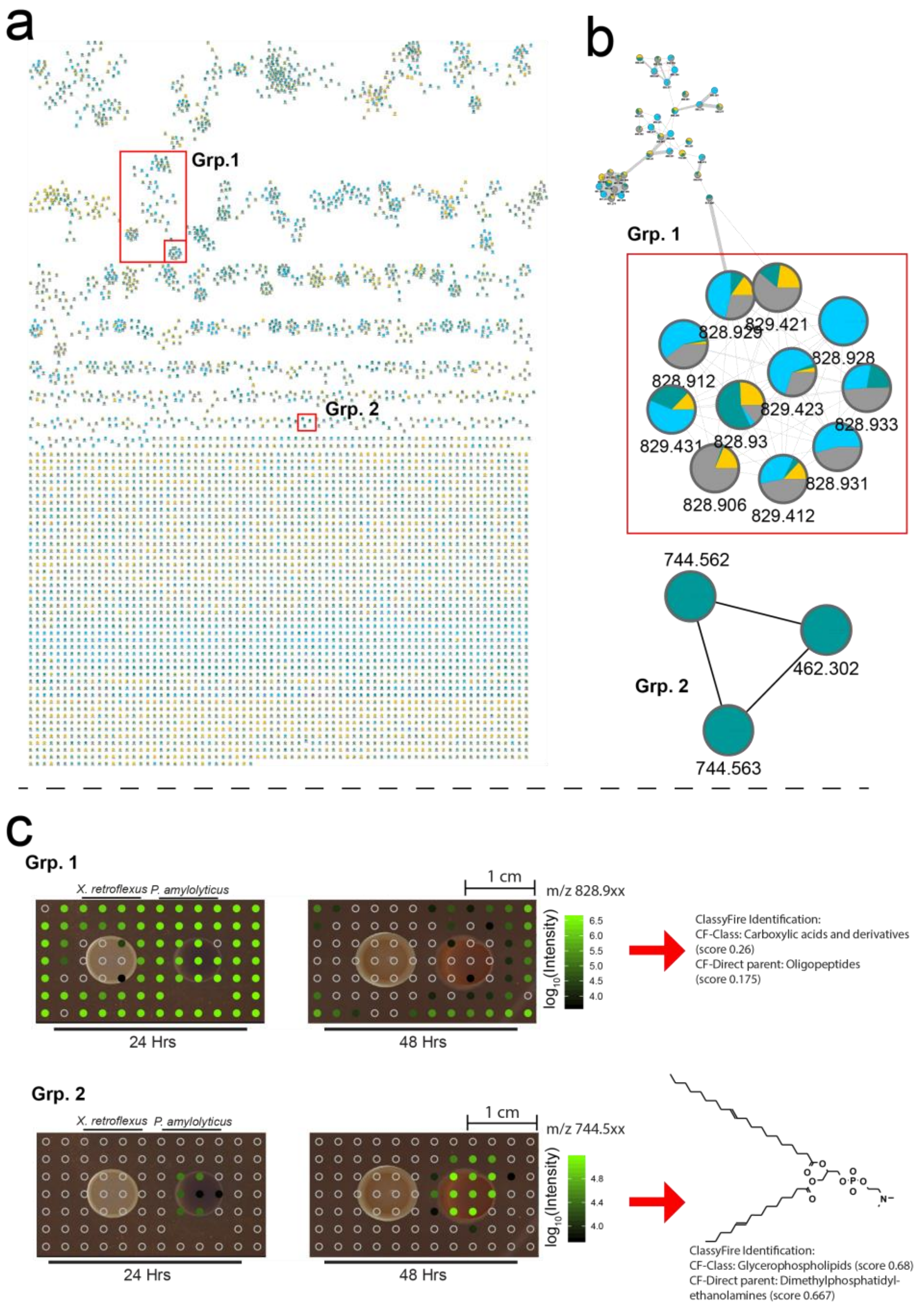

Supplementary Figure S6:

Metabolomic profiling of the interaction zone between *X. retroflexus* and *P. amylolyticus* colonies. a) Global mass spectral molecular network of the interaction zone between *X. retroflexus* and *P. amylolyticus*. Pie charts depict the mean total ion current observed per sample group: blank control agar (grey), dual species interaction zone (green), *X. retroflexus* (yellow), *P. amylolyticus* (blue). b) Schematic overview of two molecular families (two or more connected components of a graph). Molecular family 1 comprises mass spectral molecular features common to all sample groups, whereas molecular family 2 is uniquely found in the dual-species interaction zone. c) Spatial distribution of mass spectral molecular features found in molecular family 1 and 2. Circles indicate sampling points with associated MS2 data, with empty grey circles referring to spots where the molecule was not detected and filled spots where the molecule was detected. Three data files were lost during analysis, which corresponds to the blank area without circle in the interaction zone. Spot coloration from black to green corresponds to Log_{10}(MS1 ion intensity). The mass spectral molecular feature in the molecular family 1 is detected across the entire interaction zone at 24 hrs, but likely due to metabolic activity of *X. retroflexus* only in *P. amylolyticus* at 48 hrs. *In silico* structure annotation putatively identified this molecule as oligopeptide. The mass spectral molecular feature in the molecular group 2 is only detected in *P. amylolyticus* at increasing intensity from 24 to 48 hrs. *In silico* structure annotation putatively identified this molecule as a glycerophospholipid.

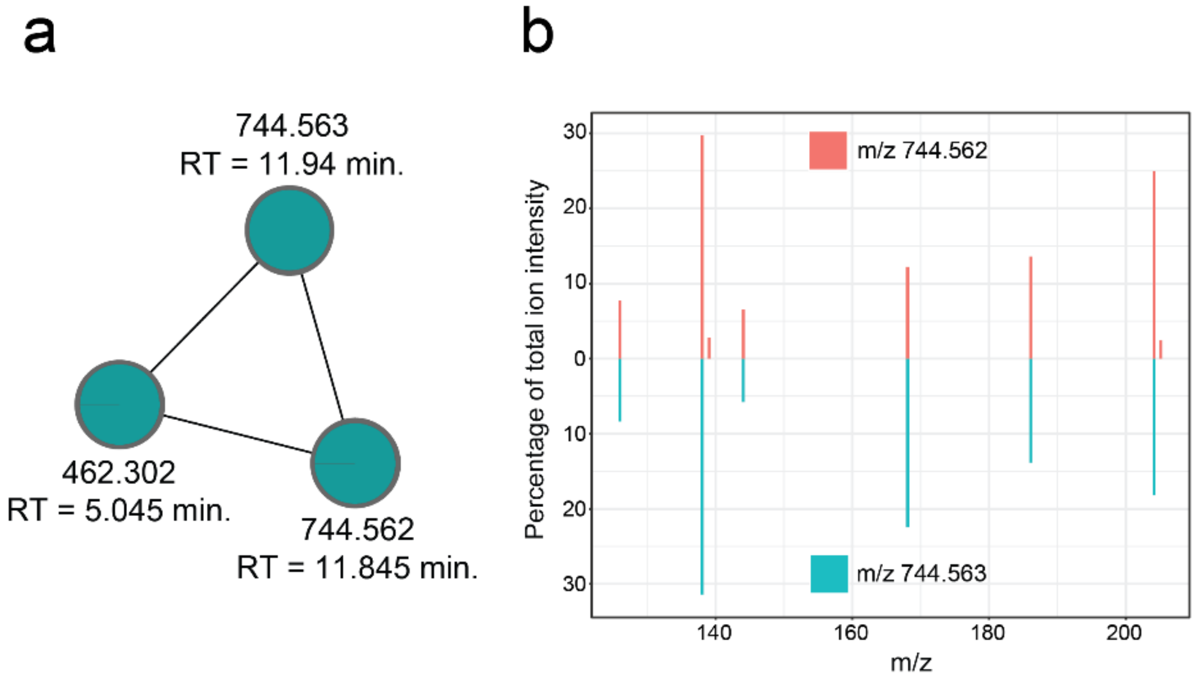

Supplementary Figure S7:
Comparison of parent mass, retention time and MS2 spectra prior to node collapse.
The two nodes m/z 744.563 and 744.562 are compared, as these two nodes are believed to correspond to the same metabolite. The parents mass of these two ions are very similar indicating that they correspond to the same metabolite. The retention time (RT) of these two ions is also very similar. Comparison of the MS2 spectra of these two ions clearly indicate their similarity as all major peaks are overlapping between the two ions. Hence, prior to visualization, ion intensities across samples are summed for these two ions.

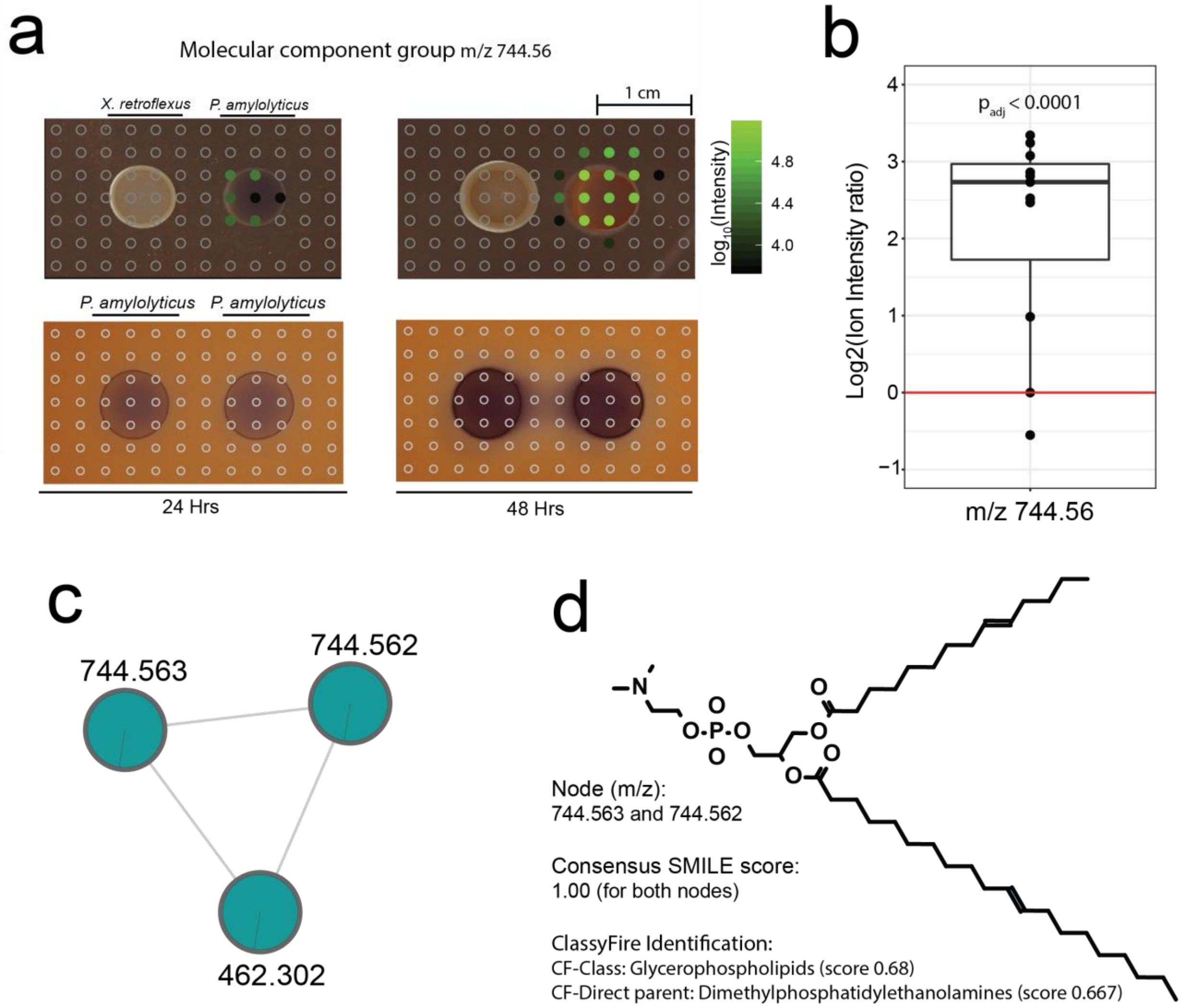

Supplementary Figure S8:

Metabolite identification and distribution of node m/z 462.302.

a) Visual distribution of node m/z 462.302. Circles indicate sampling points with associated MS data, with empty grey circles referring to spots where the metabolite was not detected and filled spots refers to spots where the metabolite was detected. Three data files were lost during analysis, which corresponds to the blank area on without circle in the interaction zone. Spot coloration from black to green refers to Log_{10}(MS1 ion intensity) of the metabolite in spots where the metabolite was detected

b) Ion intensity plot of the spots covering the 48 hrs colony of the interacting *P. amylolyticus*, as compared to the non-interacting 48 hrs colony. For the interacting colony, MS1 ion intensities were divided by 6 to account for an exponential increase in molecule production of the approximate 2.5 higher level of cells observed in the colony. For blank spots on the interacting and non-interacting colony, a threshold MS1 ion intensity equal to the lower 1% quantile of detected MS1 ion intensities was inferred. Otherwise, the measured MS1 ion intensity from the spot was used. Statistical significance was inferred by a linear model with offset set to 0 followed by FDR correction. Even when accounting for increased number of cells in the interacting colony, exponential production of the metabolite and an inferred artificial detection limit, metabolite m/z 462.302 was significantly more abundant in the interacting colony. c). Molecular cluster containing node m/z 462.302. The features from the cluster were uniquely affiliated to the dual-species sample group. d) *In silico* annotation from ClassyFire.

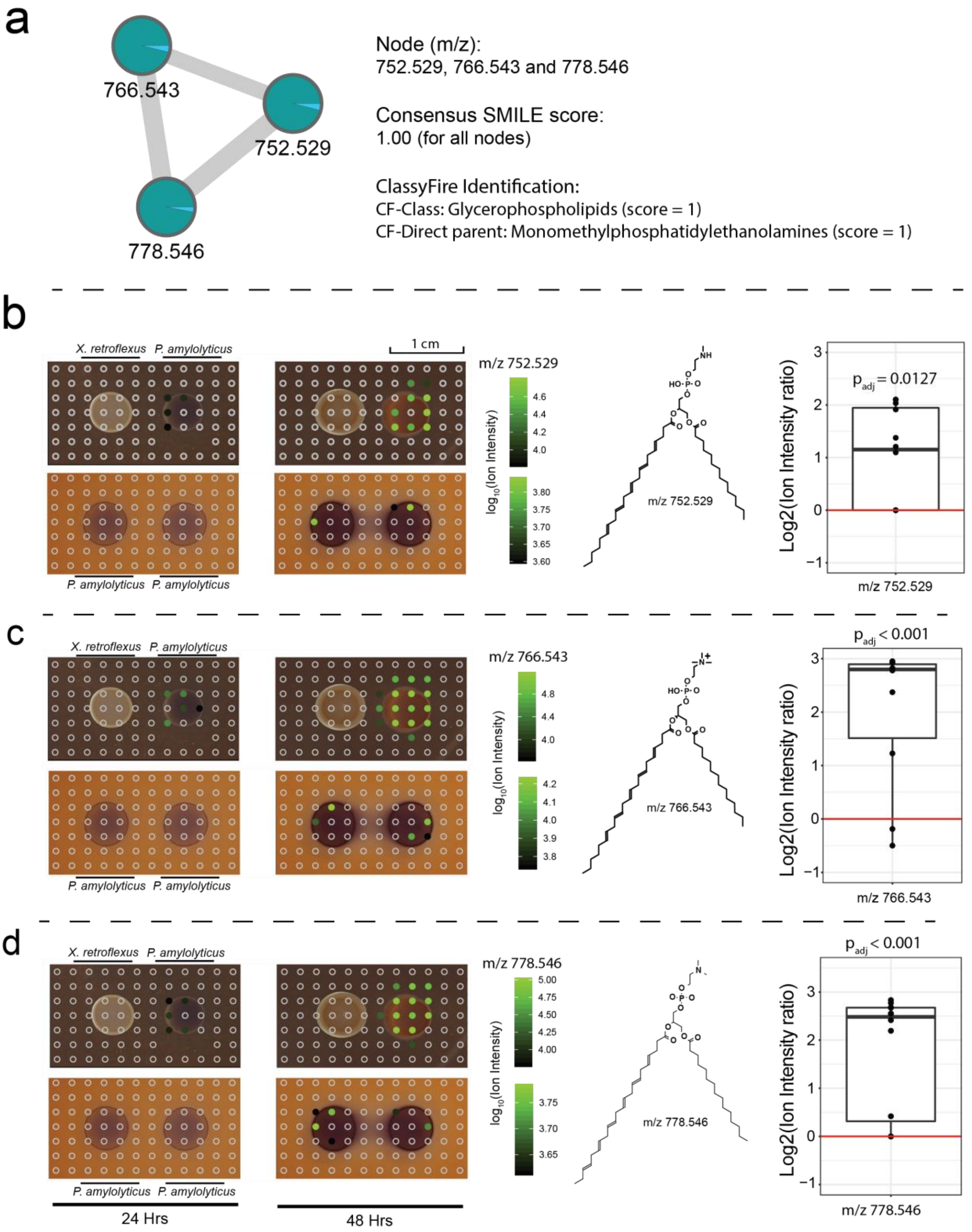

Supplementary Figure S9:

Metabolite identification and distribution of cluster with component index 113 (CI-113). a) Molecular cluster of the three nodes contained in CI-113. All three features were found in the dual-species (green color) and single-species *P. amylolyticus* group (blue color). *In silico* structure prediction indicated that all three nodes corresponded to glycerolphospholipids. b,c,d) Circles indicate sampling points with associated MS data, with empty grey circles referring to spots where the metabolite was not detected and filled spots refers to spots where the metabolite was detected. Three data files were lost during analysis, which corresponds to the blank area on without circle in the interaction zone. Spot coloration from black to green refers to Log10(MS1 ion intensity) of the metabolite in spots where the metabolite was detected. Molecular distribution of nodes m/z 752.529, m/z 766.543 and 778.546, respectively. The dual-species interaction is on top and single-species *P. amylolyticus* below, along with the predicted Consensus SMILE.

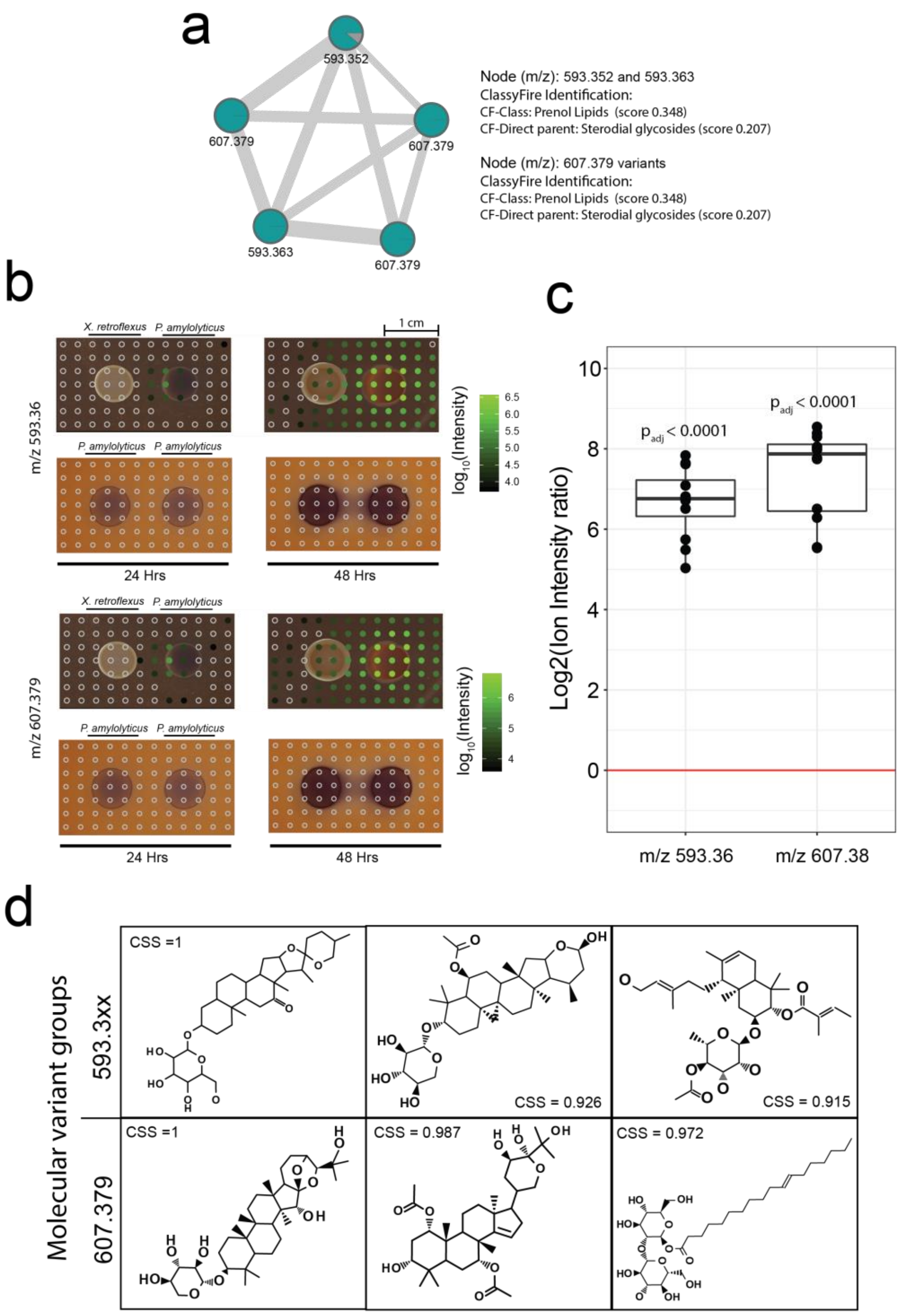

Supplementary Figure S10:

Metabolite identification and distribution of cluster with component index 159 (CI-159). a) Molecular cluster of the nodes contained in CI-159. Features were found in the dual-species (green color) and for one node in single agar control, which was believed to be a carry-over contamination from a previous sample (grey color). *In silico* structure prediction indicated that all three nodes corresponded to glycerolphospholipids. b) Molecular distributions of the collapsed molecular nodes m/z 607.379 and m/z 593xx. The nodes with parent mass m/z 607.379 were collapsed before visualization due to high similarity of parent mass, retention time and MS2 fragmentation spectra. Nodes with m/z 593.363 and m/z 593.352 were likewise collapsed. Circles indicate sampling points with associated MS data, with empty grey circles referring to spots where the metabolite was not detected and filled spots refer to spots where the metabolite was detected. Three data files were lost during analysis, which corresponds to the blank area on without circle in the interaction zone. Spot coloration from black to green refers to Log_{10}(MS1 ion intensity) of the metabolite in spots where the metabolite was detected. c) Proposed *in silico* structure for collapsed nodes m/z 607.379 and m/z 593xx. The three structures with the highest mean Consensus SMILE Score (CSS) across the Consensus SMILE belonging to each of collapsed nodes.

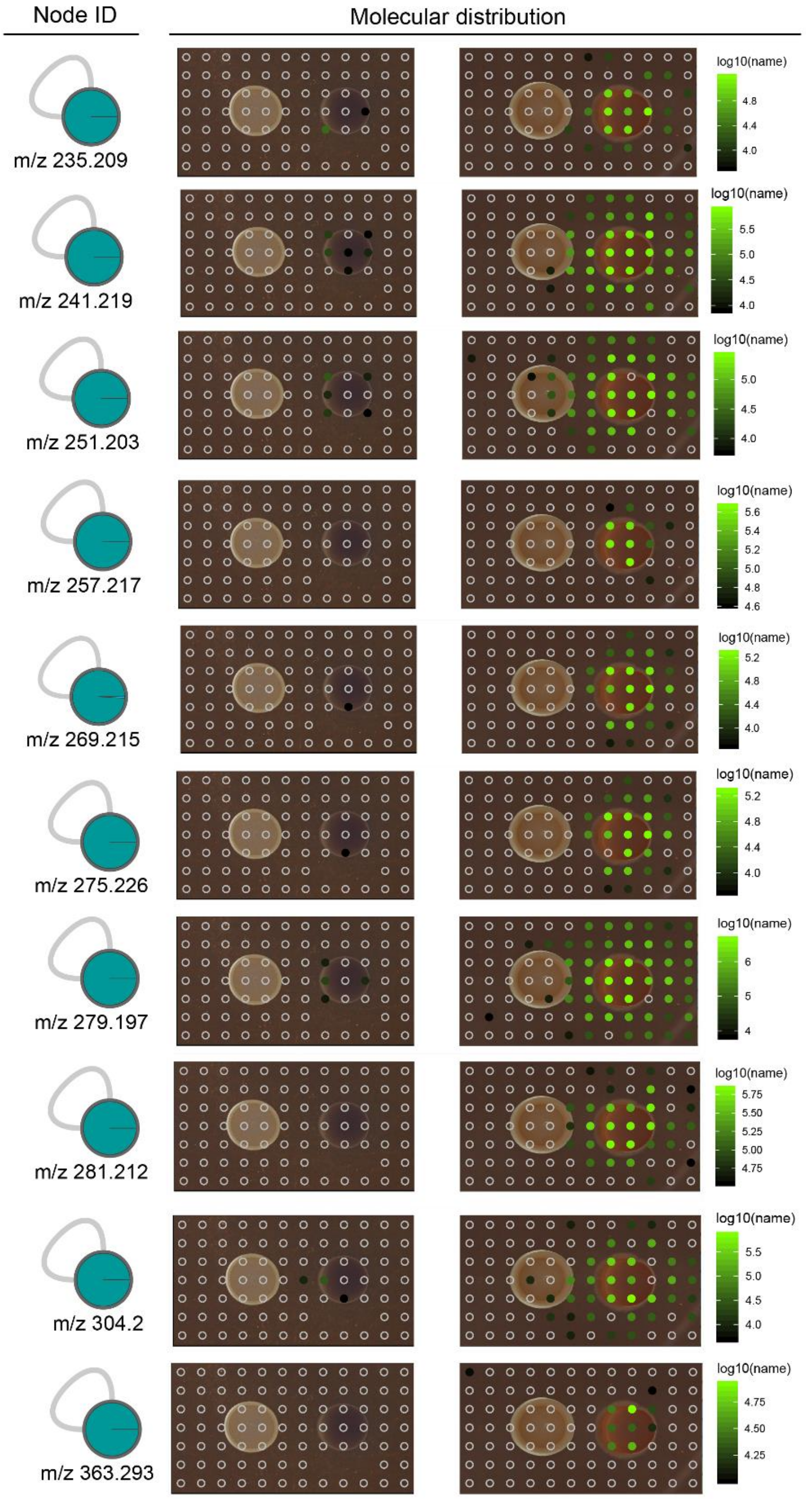

Supplementary Figure S11:
Molecular distribtuion of self-looping nodes uniquely related to the dual-species sample group with interesting visual distributions around the interacting *P. amylolyticus* colony. Set 1 of self-looping nodes.

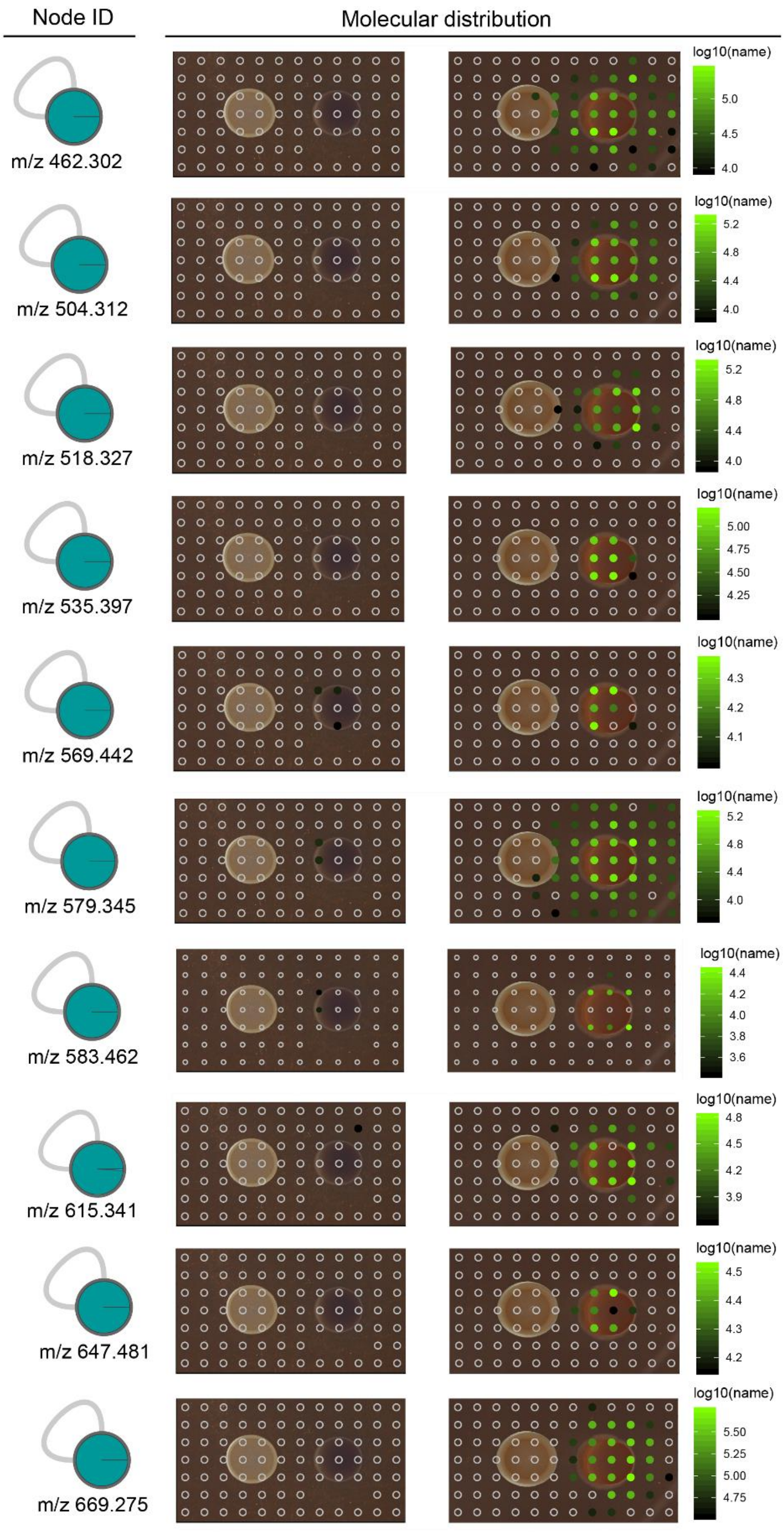

Supplementary Figure S12:
Molecular distribtuion of self-looping nodes uniquely related to the dual-species sample group with interesting visual distributions around the interacting *P. amylolyticus* colony. Set 2 of self-looping nodes.

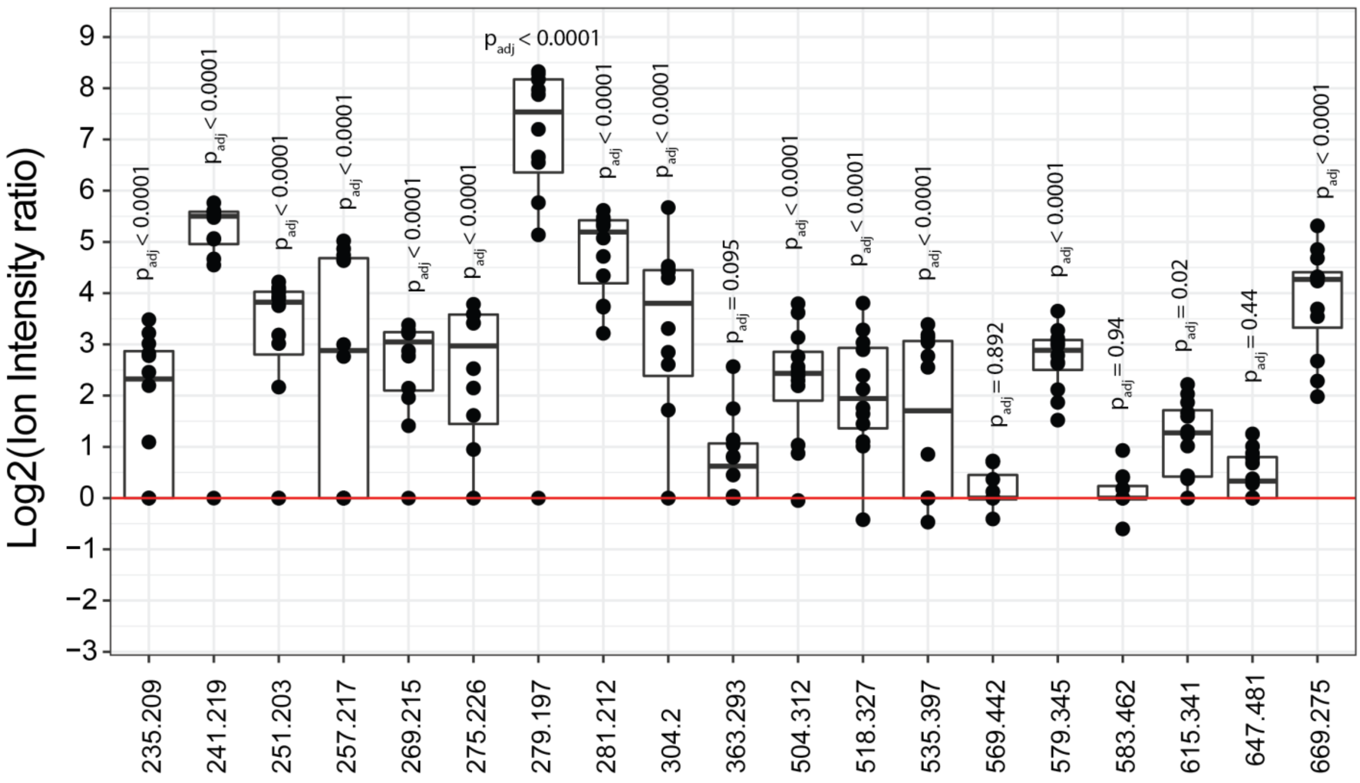

Supplementary Figure S13:
Log2 of intensity ratio from interacting *P. amylolyticus* specific MS1 molecular features, see Fig S11 and S12. MS1 ion intensities from the interacting colony were divided by 3 to account for the higher observed cell count and an inferred artificial detection limit was made for all blank observations.

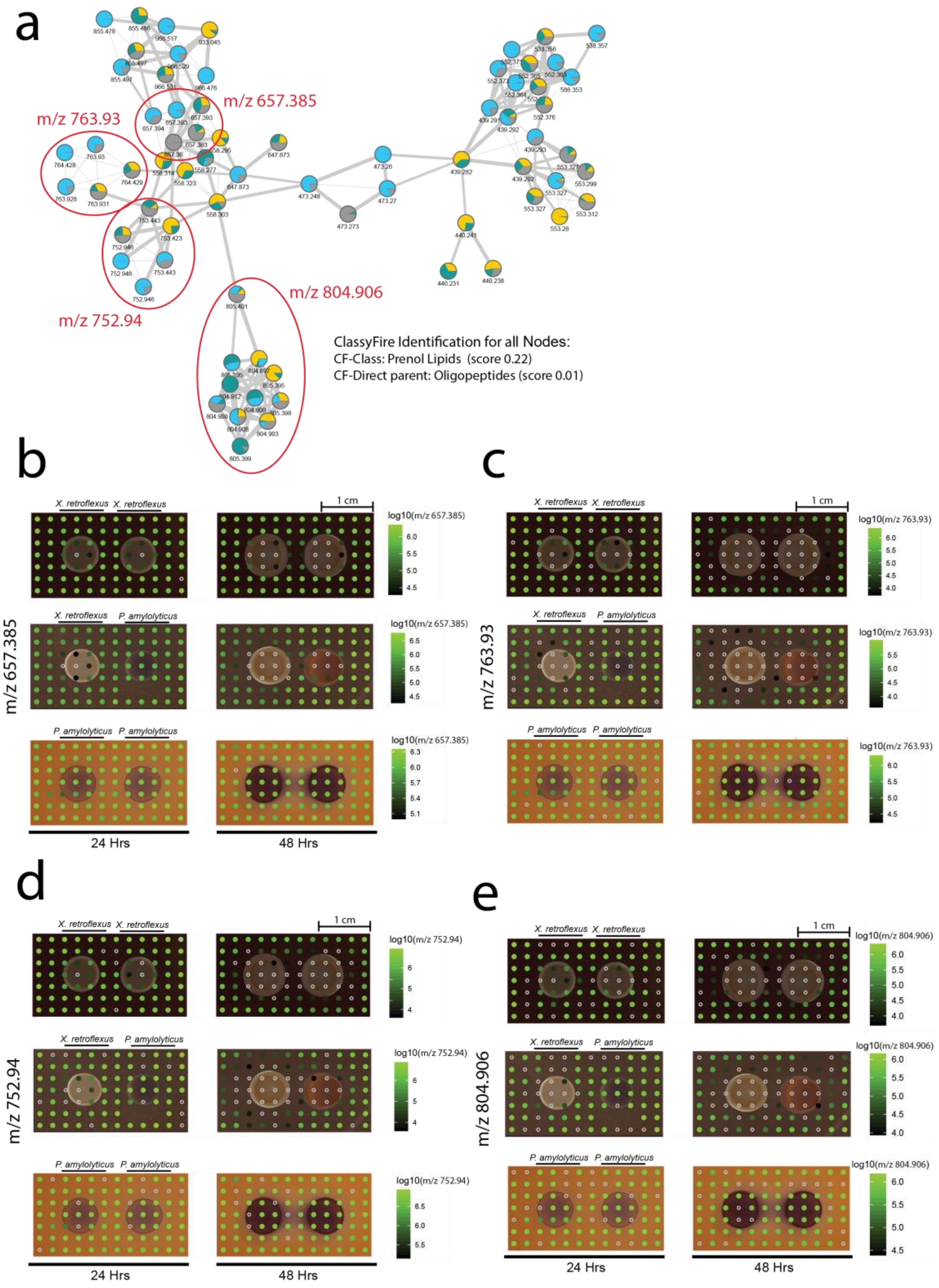

Supplementary figure S14:

Potential consumption of peptide structures from in the *X. retroflexus* controls, *P. amylolyticus* controls and dual-species interaction zone. a) Selected molecular features with specific m/z values annotated as oligo-peptides. b) spatial distribution of molecular feature with m/z 657.385 across bacterial colonies and interaction zones of *X. retroflexus-X. retroflexus, X. retroflexus-P. amylolyticus* and *P. amylolyticus-P. amylolyticus*. c) spatial distribution of molecular feature with m/z 763.93 across bacterial colonies and interaction zones of *X. retroflexus-X. retroflexus, X. retroflexus-P. amylolyticus* and *P. amylolyticus-P. amylolyticus*. d) spatial distribution of molecular feature with m/z 752.94 across bacterial colonies and interaction zones of *X. retroflexus-X. retroflexus*, *X. retroflexus-P. amylolyticus* and *P. amylolyticus-P. amylolyticus*. e) spatial distribution of molecular feature with m/z 804.906 across bacterial colonies and interaction zones of *X. retroflexus-X. retroflexus*, *X. retroflexus-P. amylolyticus* and *P. amylolyticus-P. amylolyticus*.

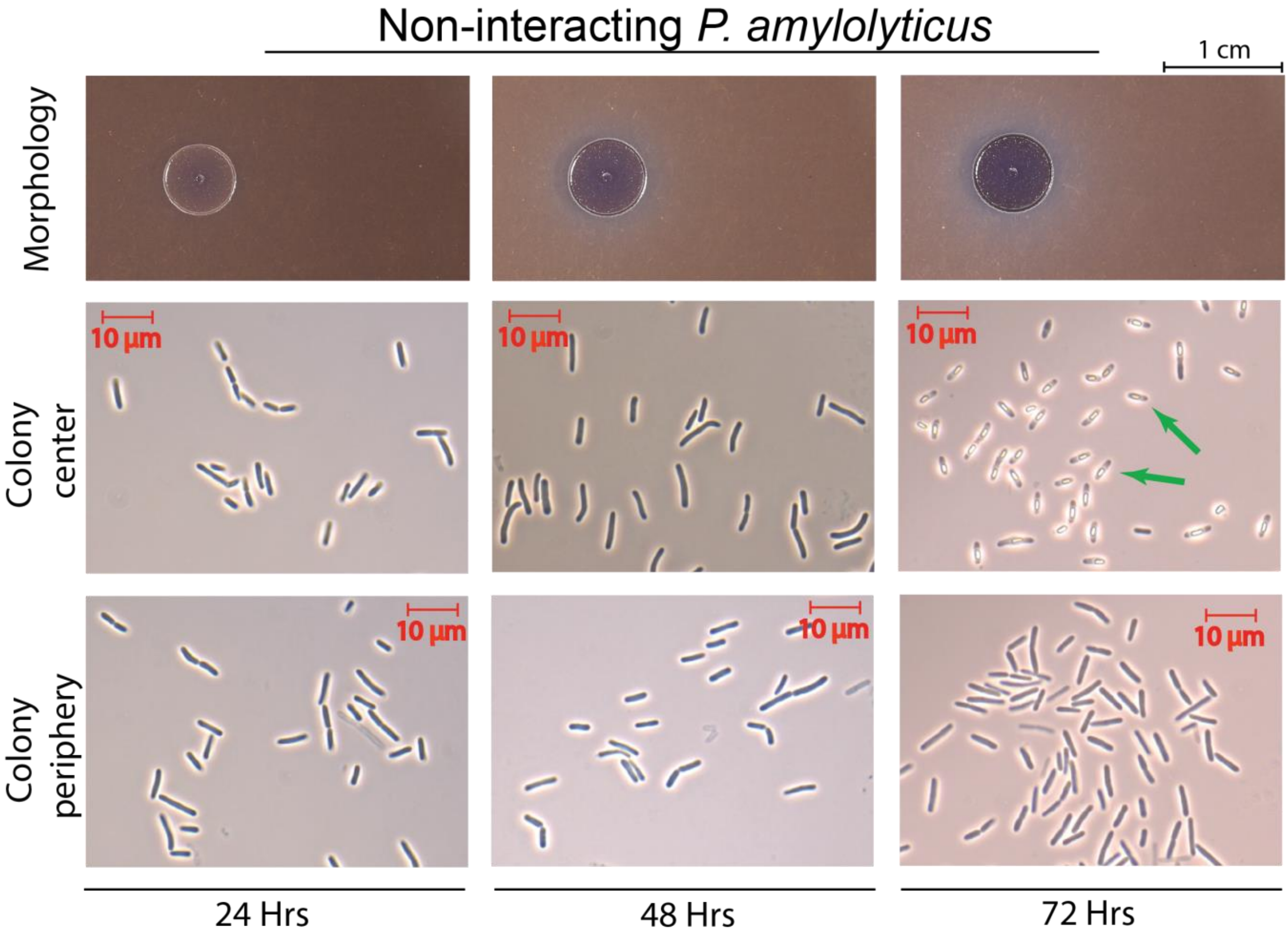

Supplementary Figure S15:
Phase contrast microscopy on non-interacting *P. amylolyticus* colony. Morphology panel shows colony morphology of a *P. amylolyticus* colony during 72 hrs of growth. Colony center and rim panels show bacterial phenotypes identified by phase contrast microscopy (63x magnification) at the colony center or rim at 24, 48 or 72 hrs. After 72 hrs, the vast majority of cell in the colony center had sporulated (indicated by green arrows). Few spores could be identified in the rim of the non-interacting *P. amylolyticus* colony at 72 hrs, but the vast majority was motile cells.

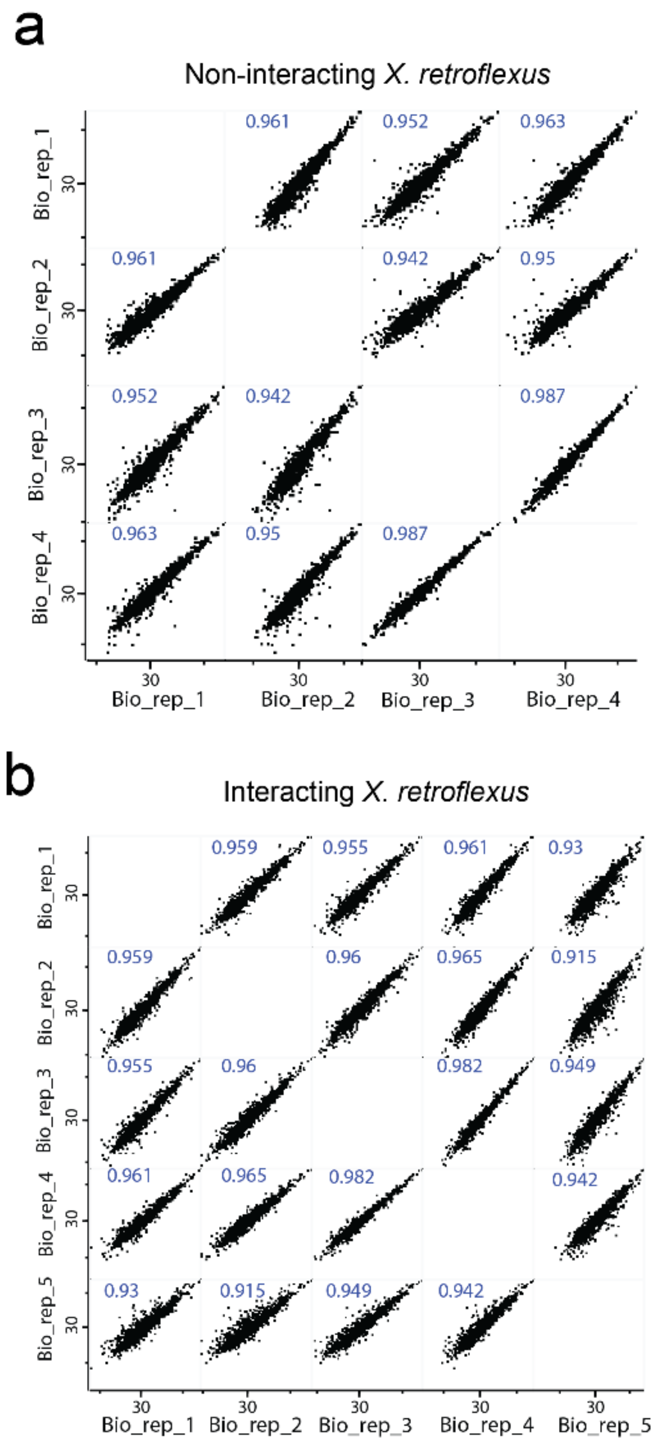

Supplementary Figure S16:

Pearson correlations of protein abundance between replicates of interacting and non-interacting *X. retroflexus.*

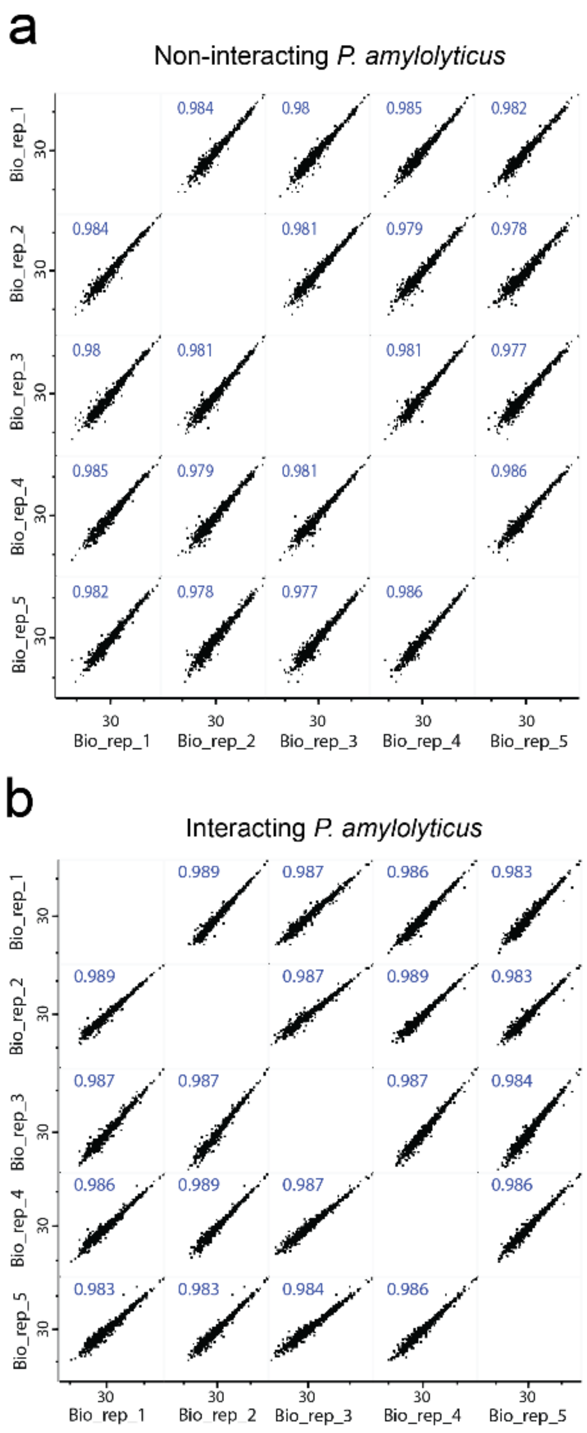

Supplementary Figure S17:

Pearson correlations of protein abundance between replicates of interacting and non-interacting *P. amylolyticus.*

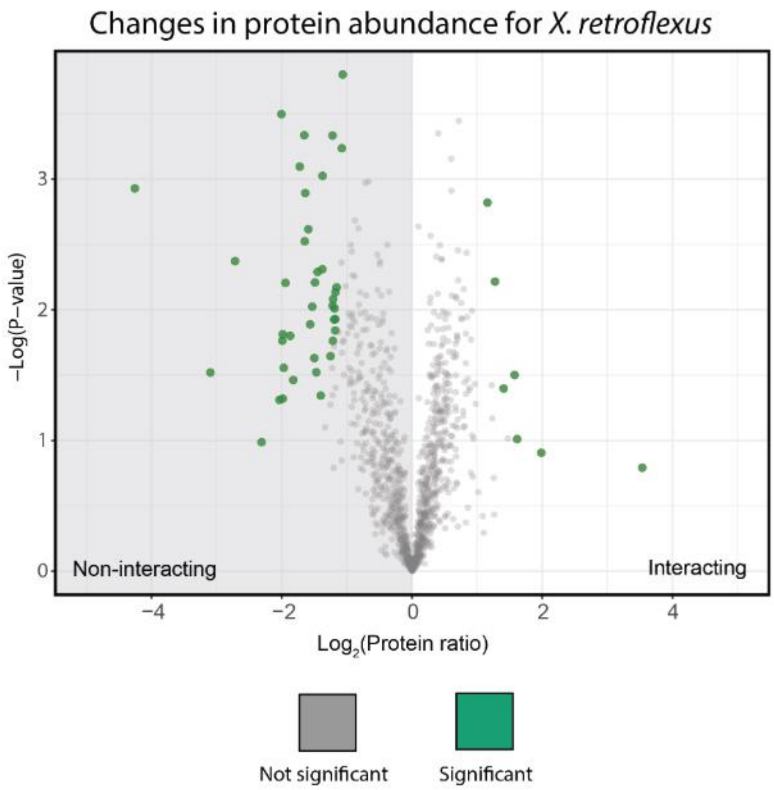

Supplementary Figure S18:
Volcano plot of the relative abundance of proteins between the interacting and the non-interacting *X. retroflexus*. Only few proteins were significantly changed in abundance between the two states, and no clear trend could be observed in terms of their associated pathways.
